# Gene S-phase kinase associated protein 2 is a novel prognostic marker in human neoplasms

**DOI:** 10.1186/s12920-023-01561-4

**Published:** 2023-06-12

**Authors:** Guo-Sheng Li, Tao Huang, Hua-Fu Zhou

**Affiliations:** 1 Department of Cardiothoracic Surgery, The First Affiliated Hospital of Guangxi Medical University, Nanning, Guangxi Zhuang Autonomous Region China; 2 Department of Cardiothoracic Vascular Surgery, The Affiliated Hospital of Youjiang Medical University for Nationalities, Baise, Guangxi Zhuang Autonomous Region China

**Keywords:** Cancer biology, Expression, Prognosis, Immunology, Biomarker

## Abstract

**Background:**

Neoplasms are a series of diseases affecting human health. Prognostic and tumor status–related markers for various tumors should be identified.

**Methods:**

Based on 19,515 samples from multiple sources, for the first time, this study provided an overview of gene S-phase kinase associated protein 2 (*SKP2*) in pan-cancer. Differential *SKP2* expression in multiple comparison groups was identified by the Kruskal–Wallis test and Wilcoxon rank-sum test. The prognosis significance of *SKP2* in individuals with neoplasm was evaluated through univariate Cox regression analysis and Kaplan-Meier curves. The area under the curve was utilized to detect the accuracy of *SKP2* in predicting cancer status. Spearman’s rank correlation coefficients were calculated in all correlation analyses. Gene set enrichment analysis was used to identify essential signaling pathways of *SKP2* in human neoplasms.

**Results:**

The study disclosed the upregulated *SKP2* expression in 15 neoplasms and decreased *SKP2* expression in three cancers (*p* < 0.05). The transcription factor Forkhead Box M1 may contribute to the increased expression levels of *SKP2* in certain tumors. Over-expressed *SKP2* represented a risk factor for the prognosis of most cancer patients (hazard ratio > 1, *p* < 0.05). *SKP2* expression made it feasible to distinguish neoplasm and control tissues of 21 neoplasms (sensitivity = 0.79, specificity = 0.87, area under the curve = 0.90), implying its potential in screening a series of neoplasms. Further, the research revealed the close association of *SKP2* expression with DNA methyltransferases, mismatch repair genes, microsatellite instability, tumor mutational burden, neoantigen count, and immunity.

**Conclusions:**

*SKP2* plays an essential role in multiple neoplasms and may serve as a marker for treating and identifying these neoplasms.

**Supplementary Information:**

The online version contains supplementary material available at 10.1186/s12920-023-01561-4.

## Introduction

Neoplasms are a series of diseases affecting human health and are the important causes of human death worldwide. It was predicted that in 2020 alone, there would be approximately 20 million newly diagnosed neoplasm patients and about 10 million related deaths worldwide [1]. Although the clinical therapeutic effect on certain tumors has been significantly improved, the prognosis of most tumor patients is not satisfactory [1–3]. Delay in diagnosis is a fundamental reason for the poor prognosis of tumor patients, and neoplasms are frequently diagnosed at an advanced stage [4]. Efforts should therefore be focused on prognostic and tumor status–related markers for various types of neoplasms.

The gene S-phase kinase associated protein 2 (*SKP2*) is located on chromosome 5p13.2 of human chromosome 5, and its homonymous encoded protein is a member of the F-box protein family [5]. *SKP2* has significant carcinogenic characteristics due to its complex functions, such as participation in cell cycle regulation. The carcinogenic characteristics of *SKP2* have been verified in several mouse experiments [5, 6], which highlights the potential of the gene for targeted therapy. In addition, in various cancers, *SKP2* is differentially (mainly highly) expressed in cancer tissues versus control tissues. For example, elevated expression of *SKP2* and its association with poor prognosis were reported in breast and prostate cancer [7, 8]. Cell experiments have also supported the promotion of *SKP2* in cancer progression [9, 10]. Thus, current studies consistently reveal the essential roles of *SKP2* in multiple tumors. However, there are no reports focusing on *SKP2* in pan-cancer. Moreover, the potential clinical significance of *SKP2* (e.g., distinguishing tumor patients from individuals without cancer) and the immune correlation of the gene in a variety of tumors are unclear.

The current study comprehensively highlights the expression levels and clinical roles (e.g., prognosis) of *SKP2* in a series of neoplasms based on thousands of samples from several sources. Further, the associations of *SKP2* expression with DNA methyltransferases (DNMTs), mismatch repair genes (MMRGs), microsatellite instability (MSI), tumor mutational burden (TMB), neoantigen count, and immunity (e.g., filtration levels of immune cells) were also revealed, which may promote understanding of *SKP2* in pan-cancers.

## Materials and methods

### Collection and processing of datasets and cohorts

An *SKP2* mRNA expression dataset of normal organs was downloaded from GTEx [11], and the dataset included 8,671 human samples. A dataset from CCLE [12], including 457 specimens of neoplasm cell lines, was obtained from DepMap Portal. Moreover, a TCGA (The Cancer Genome Atlas) cohort of 33 kinds of neoplasms containing 9,358 neoplasm and 722 control samples was derived from the Xena database. A “TARGET-AML” dataset was also acquired from the Xena database, including 173 LAML (acute myeloid leukemia) patients; among the 173 patients, 169 were with chromosome 5 alternations, including t(3;5)(q25;q34), del5q, and monosomy 5. Given the imbalanced sample size (169 versus 4) for the TARGET-AML dataset, the synthetic minority oversampling technique (SMOTE) algorithm of the “DMwR” package was used to synthesize a new dataset (16 versus 8) to validate the results based on the TARGET-AML dataset. The expression value of each gene in the mRNA-related datasets listed above was normalized with log_2_ (*x* + 1) conversion.

A total of 134 samples (three normal lung samples were used as control groups for both the LUAD and LUSC groups; Supplementary Material 1) from THPA [13, 14] were included for validating SKP2 expression at protein levels. The inclusion criteria for these specimens were as follows: (1) immunohistochemical staining for SKP2 protein involved both the neoplasm group and the control group rather than a single neoplasm group or control group; (2) both the neoplasm group and the control group contained at least two specimens; and (3) neoplasm tissues and normal tissues were processed with the same antibody. The criteria for staining scores were: 0, 1, 2, and 3 for negative, weak, moderate, and intense staining levels of SKP2 protein, respectively, while the quantity scores were: 0, 1, 2, and 3 for none, < 25%, 75–25%, and > 75% of stained cells. The total immunohistochemical score represented the SKP2 protein levels for each sample and was evaluated by the staining intensity score and the quantity score.

### Collection of clinical information

Clinical features, containing age, gender, and AJCC (American Joint Committee on Cancer) stage, were downloaded from the Xena database for the TCGA cohort. The four kinds of prognosis information—OS (overall survival), DSS (disease-specific survival time), DFI (disease-free interval), and PFI (progression-free interval)—were also obtained in the Xena database.

### Extraction of expression data of specific genes

Transcription factors (TFs) that may upregulate *SKP2* expression were predicted from the Cistrome Data Browser [15], and ChIP-Seq (chromatin immunoprecipitation followed by sequencing) data of certain TFs were also downloaded from this database. The mRNA expression data of TFs were extracted from the TCGA cohort. The expression levels of three DNMTs (DNMT1, etc.), five MMRGs (MLH1, etc.), and 46 immune checkpoints (BTLA, etc.) were also extracted in the TCGA cohort.

### Collection of other data

All the MSI, TMB, and immune neoantigen count data for patients in the TCGA cohort were downloaded from SangerBox (3.0) [16]. Immune cell infiltration levels and three kinds of scores—stromal score, immune score, and ESTIMATE score—for individuals in the TCGA cohort were downloaded from TIMER [17] and SangerBox (3.0), respectively, and the data were calculated via the corresponding TIMER and ESTIMATE algorithms.

A gene set called “c2.cp.kegg.v7.4.symbols” was downloaded from MSigDB (v7.5.1) for exploring the underlying mechanisms of *SKP2* in multiple neoplasms in terms of KEGG (Kyoto Encyclopedia of Genes and Genomes) [18–21] signaling pathways.

CRISPR (clustered regularly interspaced short palindromic repeats) data downloaded from DepMap Portal were used to explore whether *SKP2* was an essential gene for a series of cancer cells. The gene effect was utilized to evaluate the possibility of *SKP2* as an essential cancer gene — the lower the gene effect score, the higher the likelihood that *SKP2* was essential for cancer cell lines (zero scores suggesting not essential).

The half-maximal inhibitory concentration (IC50) data of 57 drugs approved by the American Food and Drug Administration or verified by clinical trials were obtained from CellMiner [22]. The data were applied to explore the susceptibility of certain drugs for patients with distinct *SKP2* expression levels and to identify potential drugs for treating these individuals.

### Statistical analysis

The chi-square test was used to detect the equilibrium of baseline data between comparison groups (e.g., cancer group versus control group). The difference in SKP2 expression in various compared groups was identified via the Kruskal–Wallis test and Wilcoxon rank-sum test. Multiple comparisons were also performed using the false discovery rate method to assess whether there was a significant difference in *SKP2* mRNA expression between neoplasms and control groups. The Wilcoxon rank-sum test was used to compare the expression levels of TFs between cancer and control groups, and Spearman’s rank correlation coefficients were utilized to evaluate the relationship between TF expression and *SKP2* expression.

The prognosis effect of *SKP2* for neoplasms patients was assessed by univariate Cox regression analysis and Kaplan-Meier curves. The area under the curve (AUC) was applied to detect the accuracy of *SKP2* in predicting cancer status. Spearman’s rank correlation coefficients were calculated in all correlation analyses. Gene set enrichment analysis (GSEA) was applied for searching KEGG pathways based on the clusterProfiler package [23]. Except for the summary receiver operating characteristic curve, which was generated in Stata (v15.0), all other analyses were carried out in R (v4.1.0). A *p*-value of less than 0.05 suggested statistical significance.

## Results

### Differential ***SKP2*** expression in neoplasms

**Fig. 1:**
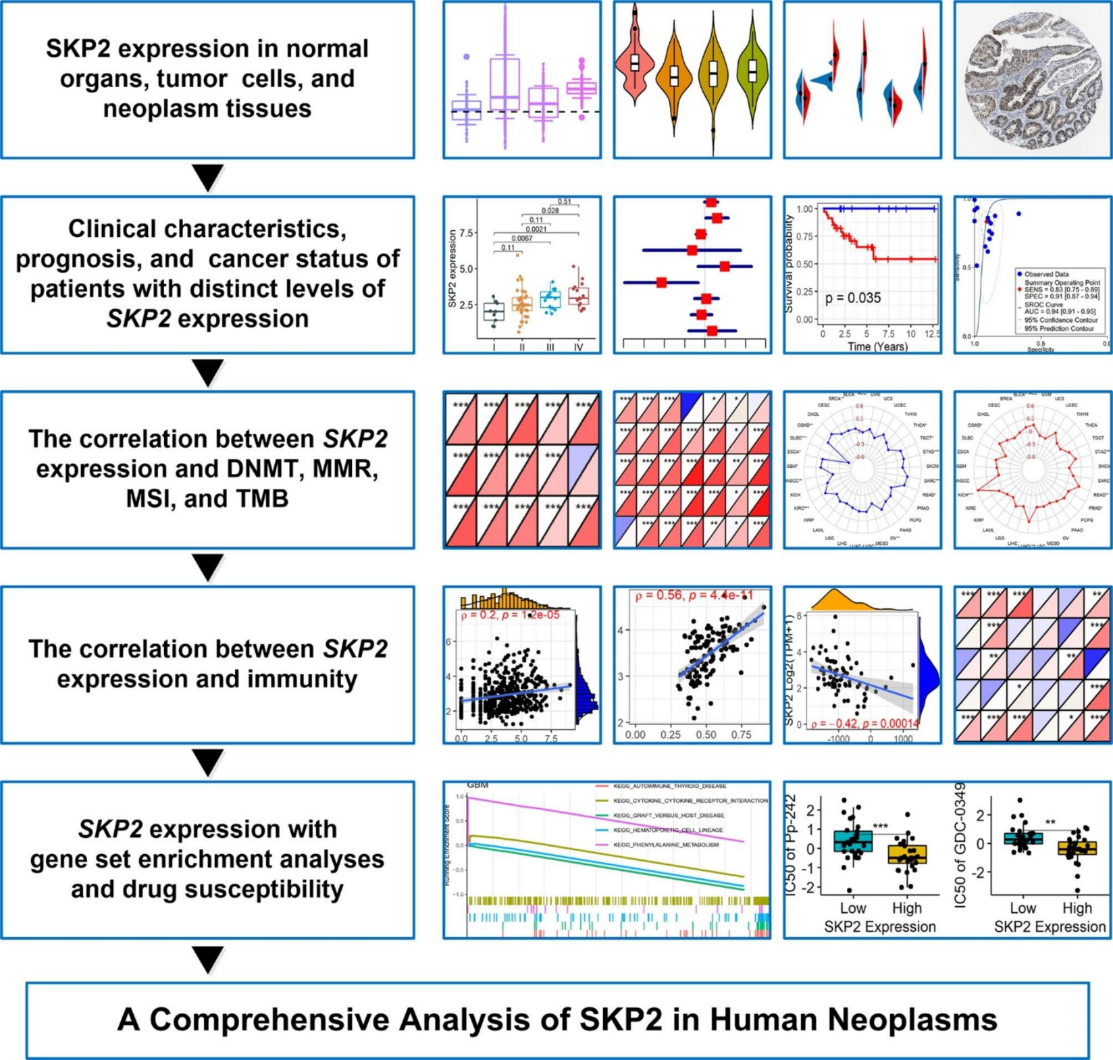
Overview of the study. DNMT, DNA methyltransferase; MMR, mismatch repair; MSI, microsatellite instability; TMB, tumor mutational burden

**Fig. 2:**
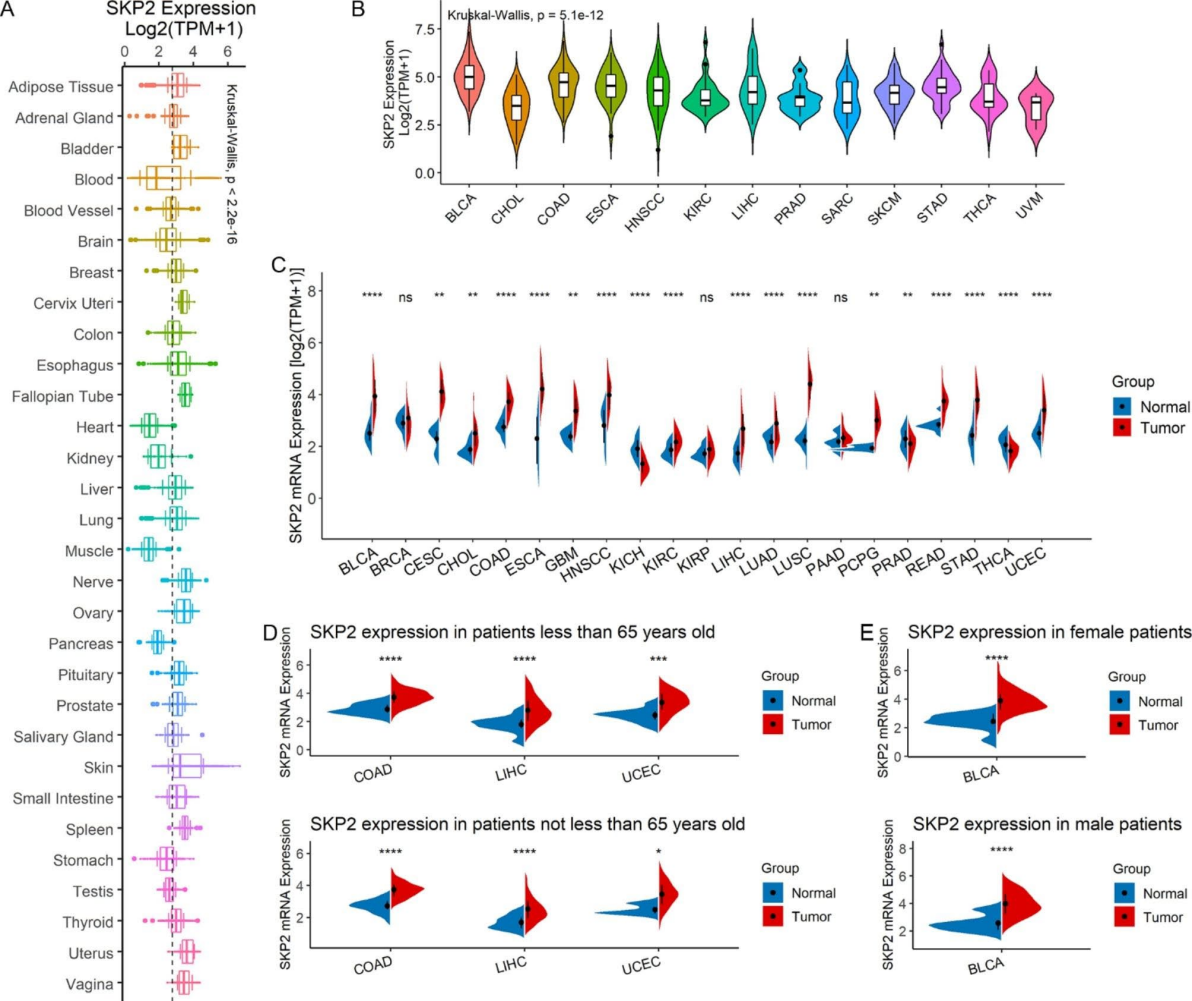
*SKP2* expression in pan-cancer and its correlations with clinical characteristics. *SKP2* expression in normal tissues (panel A), neoplasms cell lines (panel B), and pan-cancer (panels C–E). Panels D–E: Adjustment analysis of *SKP2* expression in certain neoplasms. For panels C–E: ^*^p < 0.05, ^**^p < 0.01, ^***^p < 0.001; *p*-value is based on the Wilcoxon rank-sum test (multiple comparison test by false discovery rate)

Figure 1 shows the overall design of this study. Balanced age and gender distributions were detected among the cancer and control groups for most cancers (*p* > 0.05; Supplementary Materials  2 and 3). Various organs of humans were detected with distinct *SKP2* expression levels (*p* < 0.05); for example, the cervix uteri and heart were observed increasing and decreasing *SKP2* expression levels, respectively (Fig. 2A). Similarly, differential *SKP2* expression was detected in different cell lines of neoplasms (*p* < 0.05) (Fig. 2B), and such a phenomenon was also found in tissues of 18 types of 21 investigated neoplasms — high *SKP2* expression in 15 neoplasms (bladder urothelial carcinoma [BLCA], etc.) and low *SKP2* expression in three neoplasms — KICH (kidney chromophobe), PRAD (prostate adenocarcinoma), and THCA (thyroid carcinoma) (*p* < 0.05) (Fig. 2C).

Considering the imbalanced age distribution between COAD (colon adenocarcinoma), LIHC (liver hepatocellular carcinoma), and UCEC (uterine corpus endometrial carcinoma) and their control groups (*p* < 0.05; Supplementary Material 2), adjustment tests were carried out. The results showed that, for both the old (≥ 65 years old) and the young (< 65 years old), *SKP2* expression levels in the COAD, LIHC, and UCEC groups were higher than those in their control groups (*p* < 0.05; Fig. 2D), consistent with the results without adjustment tests (Fig. 2C). The gender distribution between BLCA and its control was also imbalanced (*p* < 0.05; Supplementary Material 3), while the further adjustment test supported an increased *SKP2* expression level in the BLCA group compared to its control group (*p* < 0.05; Fig. 2E). Therefore, compared with the control tissues, the mRNA expression of *SKP2* in most cancers was significantly different.

**Fig. 3:**
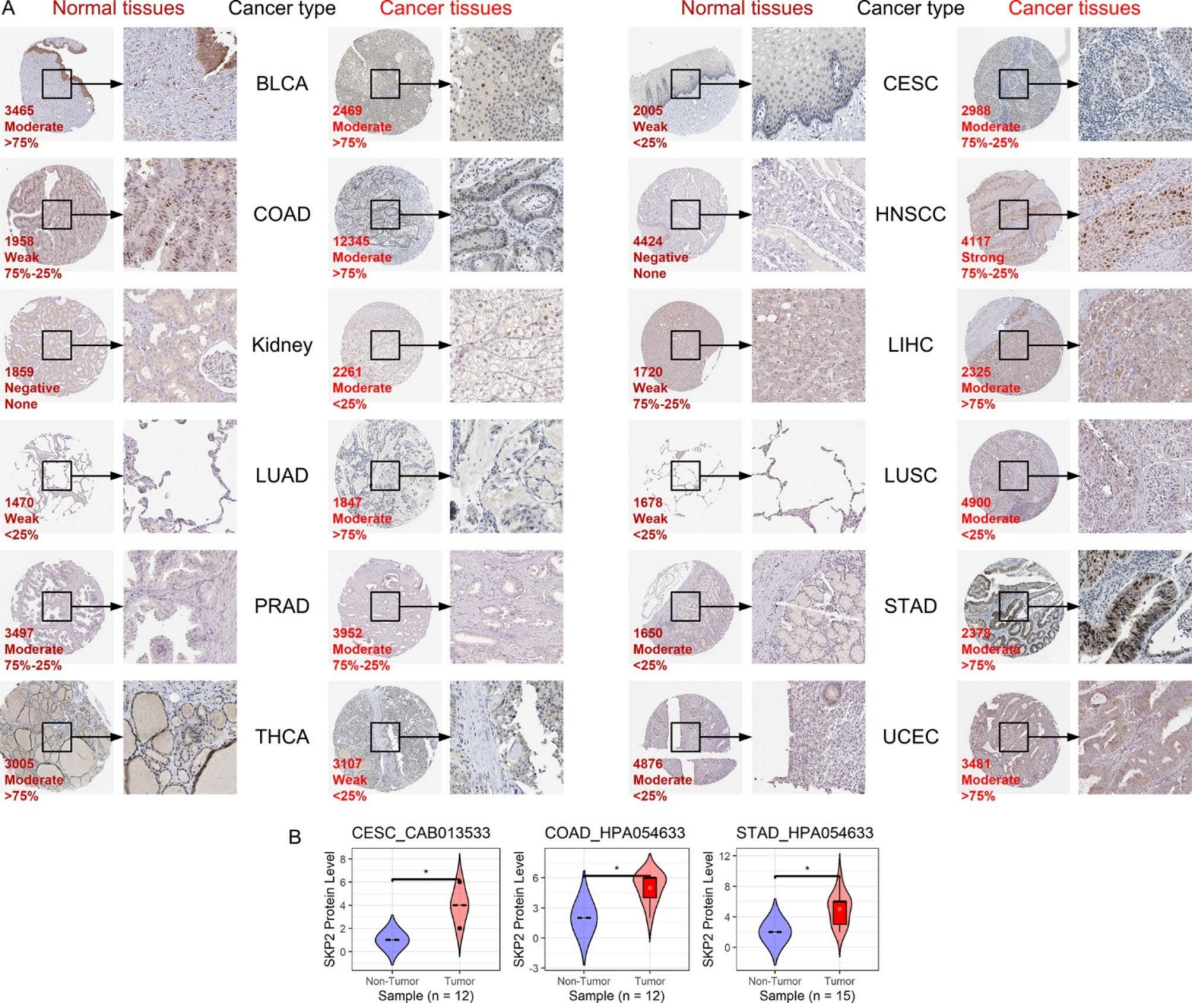
Validation of SKP2 expression in neoplasms at protein levels. Panel A: Elevated SKP2 protein levels were detected in most neoplasm (except BLCA, PRAD, and THCA) tissues rather than corresponding normal tissues; images are available from v21.0.proteinatlas.org. The three numbers at the lower left of each panel indicate patient identify document, staining level, and quantity, respectively. Panel B: Elevated SKP2 protein levels can be detected in CESC, COAD, and STAD; ^*^p < 0.05; *p*-value is based on the Wilcoxon rank-sum test

In addition to mRNA level, we evaluated the expression of SKP2 in various cancers and their control tissues at the protein level. For CESC (cervical squamous cell carcinoma and endocervical adenocarcinoma), COAD, HNSCC (head and neck squamous cell carcinoma), kidney cancer, LIHC, LUAD (lung adenocarcinoma), LUSC (lung squamous cell carcinoma), STAD (stomach adenocarcinoma), and UCEC, in comparison to normal tissues, the staining intensity of anti-SKP2 antibody was significantly stronger in cancer tissues (Fig. 3A), while the converse trend was investigated in THCA (Fig. 3A). Moreover, increasing SKP2 protein levels in CESC, COAD, and STAD were confirmed by statistical analysis (*p* < 0.05; Fig. 3B), consistent with the results at the mRNA level. Although no significant differences in SKP2 protein levels were found between cancer tissues and control tissues for the other nine neoplasms (e.g., BLCA), the expression of SKP2 at both protein and mRNA levels exhibited the same trends for most of these neoplasms (Supplementary Material 4).

According to the above results, *SKP2* was upregulated in up to 15 cancers discussed in this research. TFs tend to play essential roles in gene expression regulation. TFs that may regulate *SKP2* expression in the 15 cancers were investigated, which may contribute to understanding possible mechanisms for the overexpression of *SKP2*. A total of 200 TFs that may upregulate *SKP2* expression were obtained from the Cistrome Data Browser. Among these TFs, FOXM1 (forkhead box M1) is encoded by the gene *FOXM1*. *FOXM1* was highly expressed in the 15 cancers (*p* < 0.05; Supplementary Material 5A), where elevated *SKP2* expression was also detected. Moreover, significantly positive correlations were found between the expression levels of *FOXM1* and *SKP2* (*ρ* ≥ 0.3, *p* < 0.05; Supplementary Material 5B). What is important is that the binding peaks of FOXM1 can be observed upstream of the transcription start site of *SKP2* (Supplementary Material 5C). These results indicated that elevated FOXM1 expression might contribute to the high *SKP2* expression in the 15 cancers.

Notably, the possible mechanisms of SKP2 expression in cancers may be complex. For example, chromosome 5 alternations (*SKP2* is located on chromosome 5) are commonly observed in LAML [24]. Compared to the group without chromosome 5 alternations, a trend of downregulation of *SKP2* expression levels can be observed in LAML patients with chromosome 5 alternations, based on the raw data (Supplementary Material 6A). The result was validated via the SMOTE data (*p* < 0.05, Supplementary Material 6B). This suggests that, except for the regulatory effect of TFs, attention should also be given to other factors that may affect SKP2 expression in specific cancers. Thus, the underlying mechanisms of SKP2 expression in cancers require further investigation.

### Clinical characteristics of patients with various ***SKP2*** expression levels

**Fig. 4:**
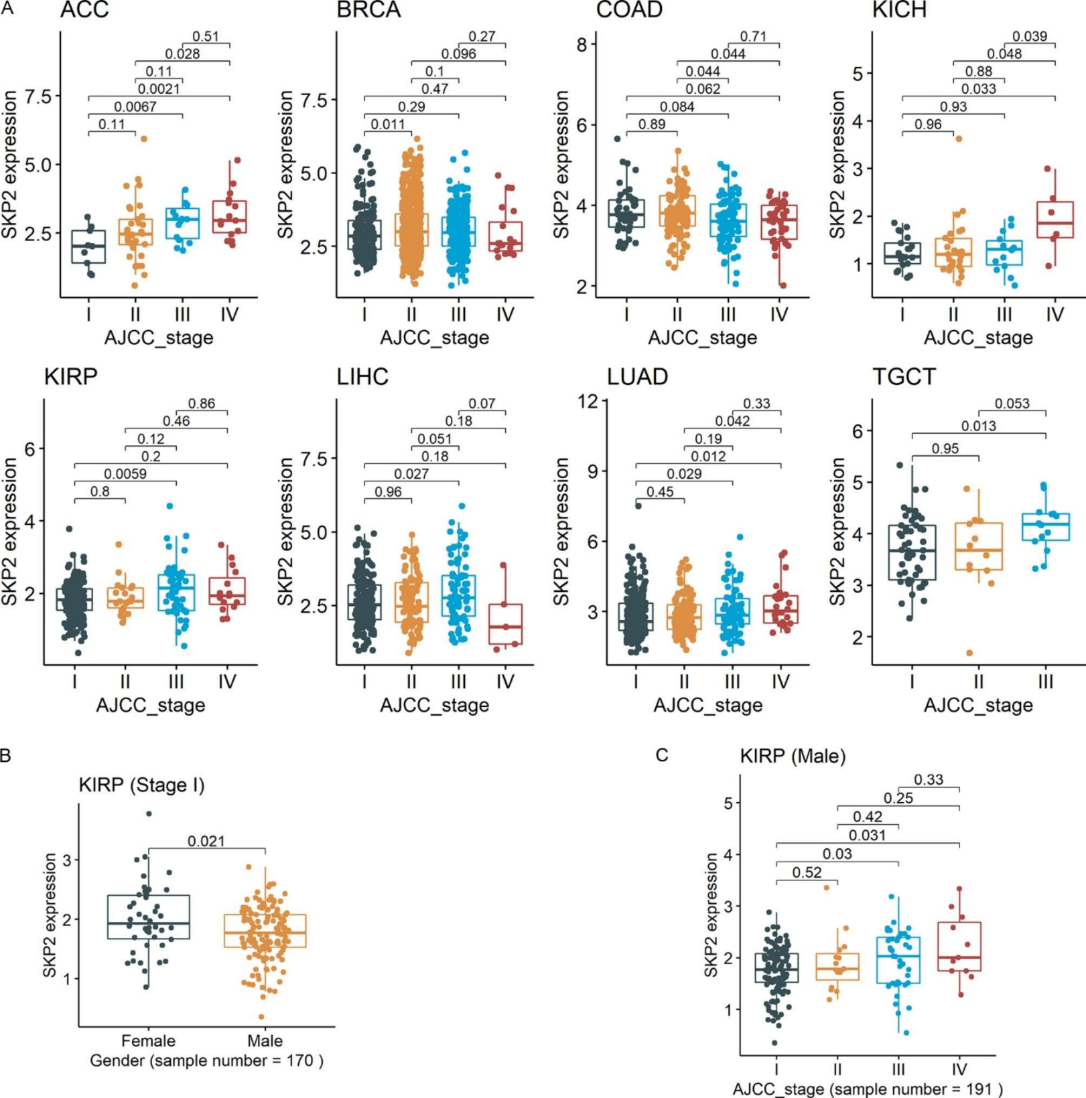
Correlations between *SKP2* expression with clinical parameters. For panels A–C, the *p*-value is based on the Wilcoxon rank-sum test

Patients with different clinical characteristics may have various prognoses. For example, cancer patients with advanced AJCC stages tend to have poorer prognoses than others. Thus, we analyzed the relationship between *SKP2* expression and available clinical characteristics data of patients. The results showed that increased *SKP2* expression tended to be observed in advanced AJCC stages for certain neoplasms (*p* < 0.05) (Fig. 4A and Supplementary Material 7). Elevated *SKP2* expression was observed in male instead of female patients with HNSCC, LAML, or READ (rectum adenocarcinoma); in contrast, the opposite phenomenon was found in KIRP (kidney renal papillary cell carcinoma) and SARC (sarcoma) (*p* < 0.05) (Supplementary Material 8). Individuals with cancer of different ages exhibited varying levels of *SKP2* expression. For instance, decreasing *SKP2* expression was also detected in older patients with BRCA (breast invasive carcinoma) and young patients with PCPG (pheochromocytoma and paraganglioma) (*p* < 0.05) (Supplementary Material 9).

Given that *SKP2* expression was associated with at least two clinical characteristics among five neoplasms (BRCA, KIRP, LIHC, LUAD, and READ), covariate analysis was performed for these neoplasms. Consequently, AJCC stage distribution was correlated with age distribution in KIRP (*p* < 0.05; Supplementary Material 10) and, thus, further adjustment analysis was carried out for this cancer. For patients with this cancer at AJCC stage I, males were observed to have lower *SKP2* expression than females (Fig. 4B); this discrepancy was not identified for KIRP at stages II, III, or IV (Supplementary Material 11). For KIRP, a positive association between *SKP2* expression and AJCC stage was determined not for females but for males (*p* < 0.05) (Fig. 4C and Supplementary Material 11).

### Prognosis differences of patients with disparate ***SKP2*** expression levels

**Fig. 5:**
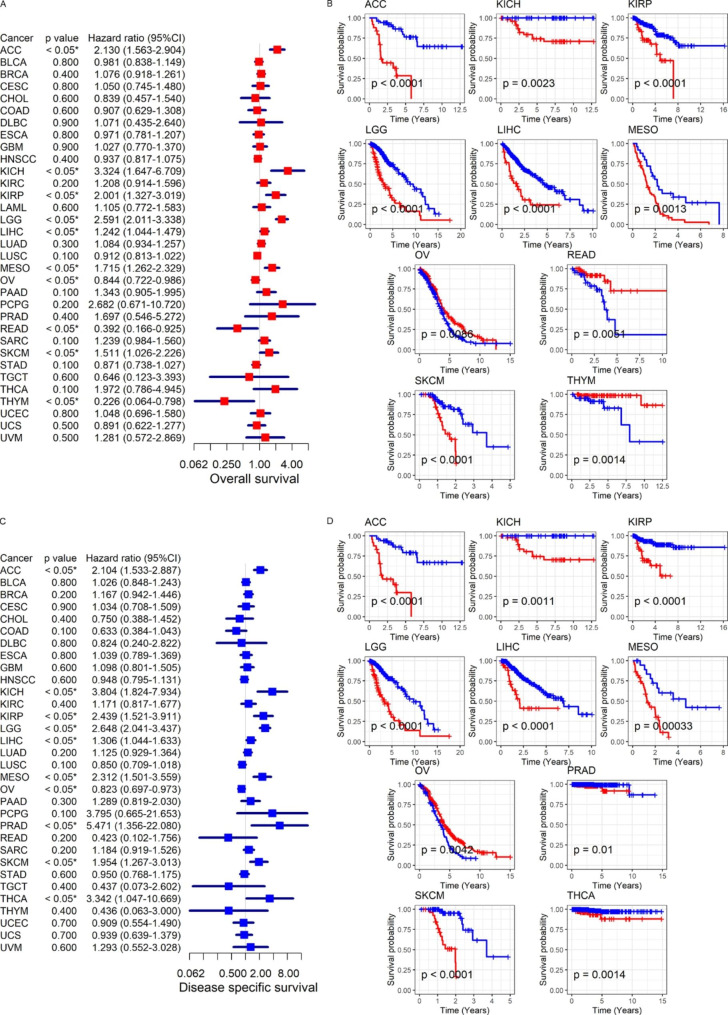
Relation of *SKP2* expression with overall survival (panels A–B) and disease-specific survival of neoplasm patients (panels C–D)

**Fig. 6:**
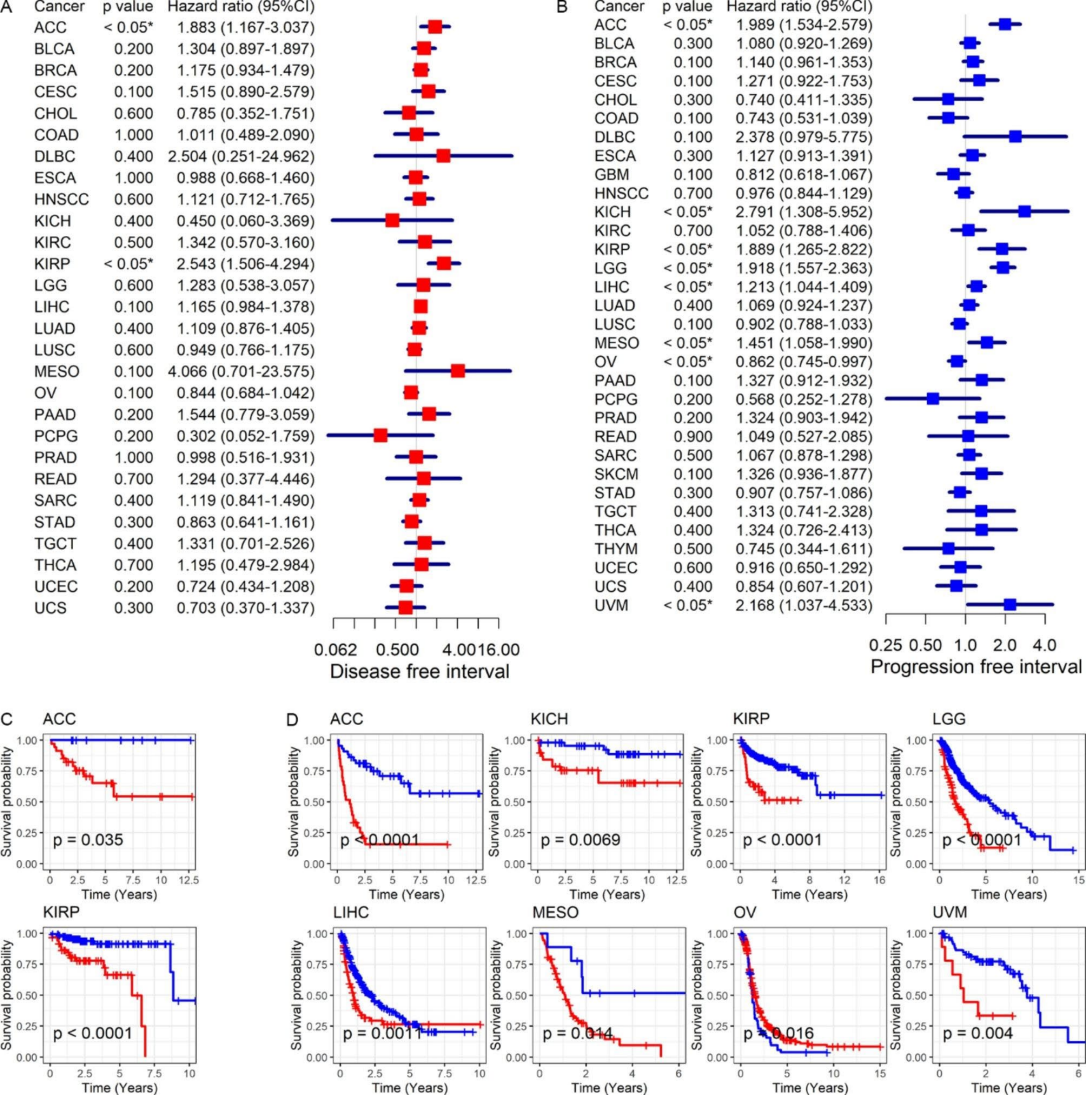
Relation of *SKP2* expression with disease-free interval (panels A–B) and progression-free interval of neoplasm patients (panels C–D)

To directly evaluate the prognostic significance of *SKP2* expression in various tumors, univariate Cox regression analyses and Kaplan-Meier curves were performed in this research. The two analysis methods consistently indicated that elevated *SKP2* expression represented poor OS and/or DSS for patients with ACC (adrenocortical carcinoma), KICH, KIRP, LGG (brain lower grade glioma), LIHC, MESO (mesothelioma), PRAD, SKCM (skin cutaneous melanoma), or THCA (hazard ratio [HR] > 1, *p* < 0.05) and favorable OS and DSS for individuals with OV (ovarian serous cystadenocarcinoma), READ, or THYM (thymoma) (HR < 1, *p* < 0.05) (Fig. 5A–D). In DFI and PFI, *SKP2* expression also played a risk role for patients with ACC and KIRP (HR > 1, *p* < 0.05) (Fig. 6A–D). Moreover, *SKP2* expression was significantly correlated with poor PFI in individuals with KICH, LGG, LIHC, MESO, or UVM (uveal melanoma) (HR > 1, *p* < 0.05), as well as with considerable PFI in OV patients (HR < 1, *p* < 0.05) (Fig. 6B and D).

### Difference in cancer status of patients with distinct ***SKP2*** expression levels

**Fig. 7:**
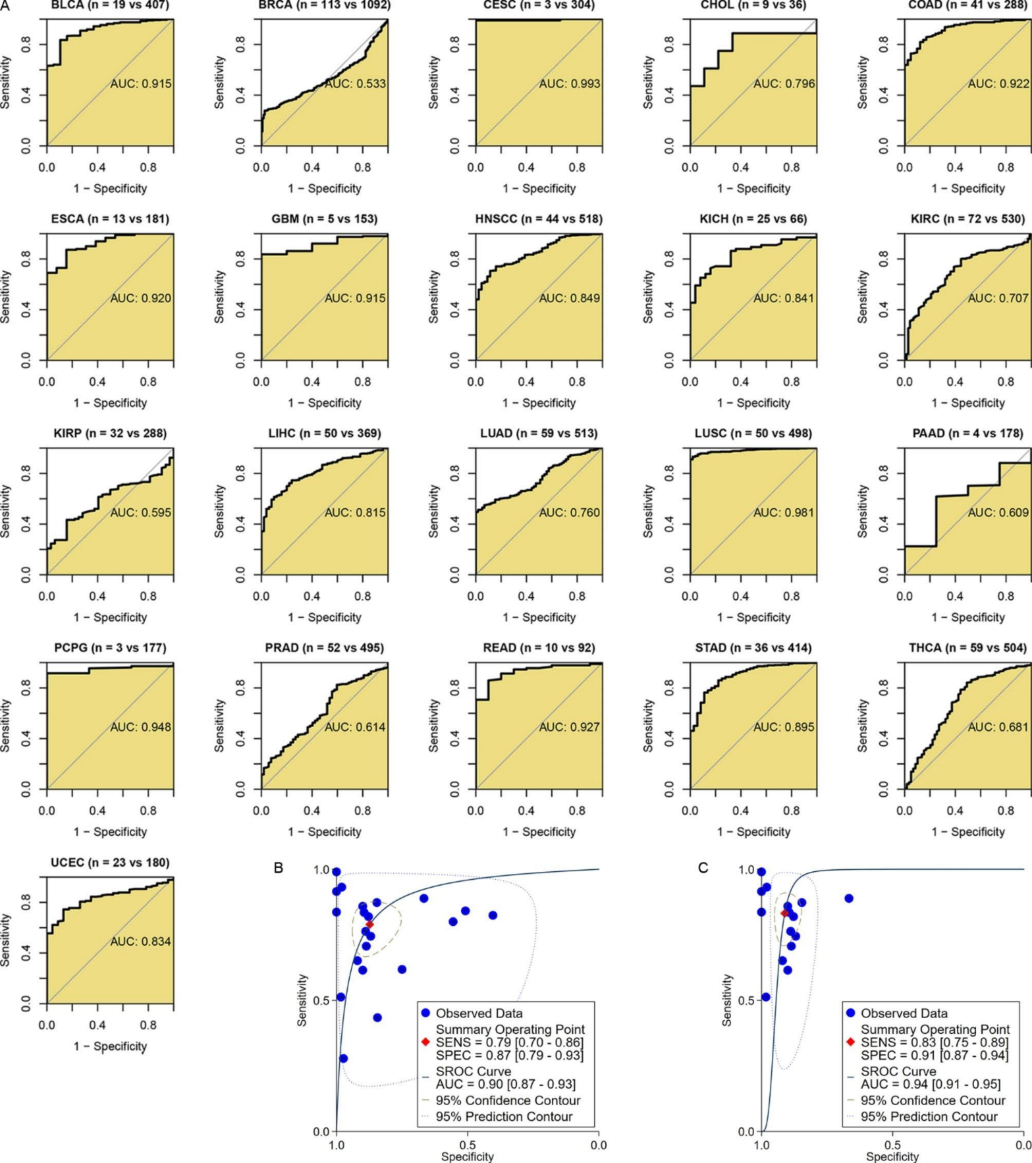
Receiver operating characteristic curves for detecting the ability of *SKP2* expression to distinguish neoplasm tissues from their normal tissues. Panel A: Receiver operating characteristic curves. Panels B–C: Summary receiver operating characteristic curves. AUC, area under the curve

The prognostic significance of *SKP2* expression in various cancers was notable, but it was still unclear whether *SKP2* could distinguish cancer samples from control samples, which was analyzed in this study. In 21 kinds of neoplasms investigated in this part, *SKP2* well differentiated 15 types of neoplasm (BLCA, etc.) tissues and their normal counterparts (AUC > 0.75) (Fig. 7A). Particularly, *SKP2* expression had a remarkable capacity to differentiate eight cancers from their control tissues; the eight neoplasms were BLCA, CESC, COAD, ESCA (esophageal carcinoma), GBM (glioblastoma multiforme), LUSC, PCPG (pheochromocytoma and paraganglioma), and READ (AUC > 0.90) (Fig. 7A). *SKP2* expression made it feasible to discern the neoplasm and control tissues of the 21 cancers (sensitivity = 0.79, specificity = 0.87, AUC = 0.90) (Fig. 7B), particularly for the 15 cancers listed above (sensitivity = 0.83, specificity = 0.91, AUC = 0.94) (Fig. 7C).

**Fig. 8:**
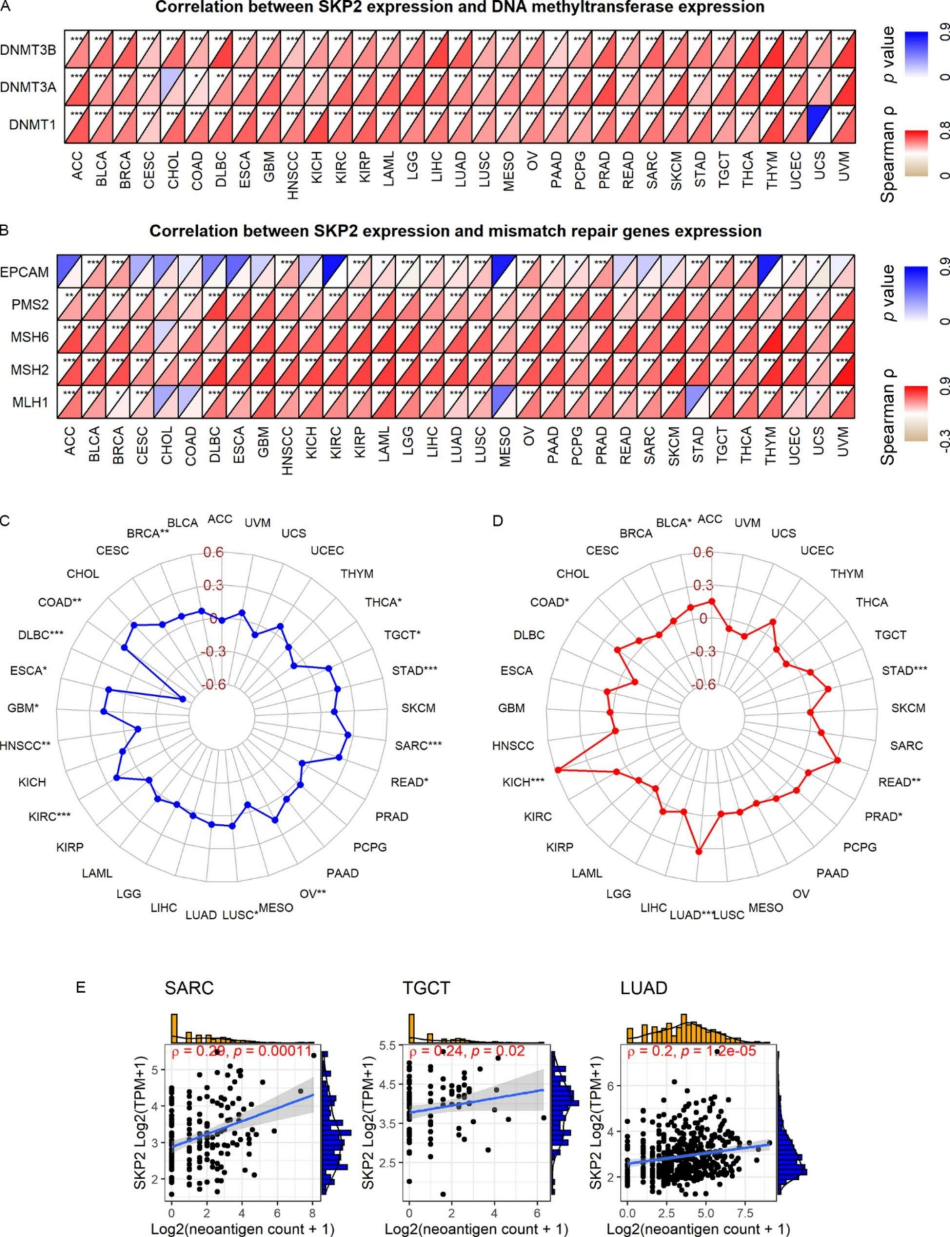
Spearman coefficient of *SKP2* expression with DNA methyltransferase expression (panel A), mismatch repair gene expression (panel B), microsatellite instability (panel C), tumor mutational burden (panel D), and immune neoantigen count (panel E). The letter “ρ” is followed by the Spearman correlation coefficient

### ***SKP2*** expression with DNMTs, MMRGs, MSI, and TMB

DNMTs, MMRGs, MSI, and TMB are considered prognostic markers and treatment indicators of cancer [25–27]. Exploring the relationship between *SKP2* and these indicators may help identify the marker role of *SKP2* in neoplasms. A significantly positive correlation between *SKP2* expression and the expression levels of three DNMTs and five MMRGs was detected in almost all the 33 cancers analyzed (Fig. 8A and B). The close association of *SKP2* expression with MSI and TMB was also observed in multiple neoplasms, especially for DLBC (lymphoid neoplasm diffuse large B-cell lymphoma) (*ρ* = −0.51, *p* < 0.05) in MSI (Fig. 8C), as well as KICH, LUAD (lung adenocarcinoma), and READ in TMB (*ρ* > 0.3, *p* < 0.05) (Fig. 8D). These results suggest that *SKP2* may affect the progression of neoplasms by influencing DNMTs, MMRGs, MSI, and TMB.

### ***SKP2*** expression with immunity

**Fig. 9:**
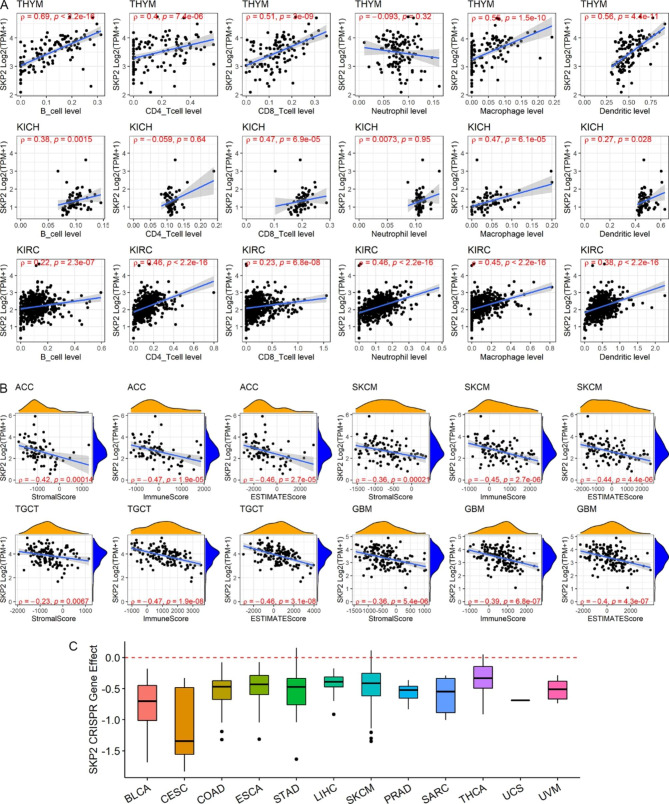
*SKP2* expression with the immune microenvironment, and its essential role in some cancer types. Panels A–B: The relevance between *SKP2* expression with infiltration levels of immune cells (panel A) and immune-related scores (panel B). Panel C: *SPK2* was identified as an essential cancer gene with a gene effect score of less than 0 in most cancers

Elevated MSI and TMB tend to increase immune neoantigen levels [28, 29]; thus, the relationship between immune neoantigen count and *SKP2* expression was investigated. As a result, the positive relevance of immune neoantigen count with *SKP2* expression was detected in SARC, TGCT (testicular germ cell tumors), and LUAD (*p* < 0.05) (Fig. 8E). The immune neoantigen can potentially induce an immune response, which involves activating a variety of immune cells. Interestingly, significant correlations (generally positive) between the *SKP2* expression and infiltration levels of most of the six immune cells — B cells, CD4 T cells, CD8 T cells, neutrophil cells, macrophage cells, and dendritic cells — were observed in THYM, KICH, and KIRC (kidney renal clear cell carcinoma) (Fig. 9A). Furthermore, *SKP2* was also closely related to the immune microenvironment, including immune cells. For instance, for ACC, SKCM, TGCT, and GBM, negative associations between *SKP2* expression and immune environment (including stromal, immune, and ESTIMATE scores) were detected (Fig. 9B). Notably, the relationship between *SKP2* expression and both immune cell infiltration levels and immune environment scores varied with tumor type (Supplementary Materials 12 and 13), implying the complex mechanisms of *SKP2* in neoplasms.

Immune checkpoints are known to regulate the growth and proliferation of immune cells negatively. In our study, *SKP2* expression was relevant to the expression levels of multiple immune checkpoints. Particularly, the close correlation (*ρ* > 0.3, *p* < 0.05) between *SKP2* expression and at least 15 immune checkpoints can be detected in DLBC, KIRC, PRAD, and UVM (Supplementary Material 14), which may partly explain the correlations between *SKP2* expression and the immune environment.

### ***SKP2*** expression with GSEA

*SKP2* plays an essential role in multiple neoplasms, and the corresponding mechanisms require investigation. Previous studies have revealed the molecular mechanism of the gene in a single neoplasm, but whether *SKP2* had a similar molecular mechanism in a variety of tumors was unknown. Therefore, we attempted to clarify this issue via GSEA. Among the 26 KEGG signaling pathways with a GSEA *p*-value less than 0.05, the “olfactory transduction” pathway was observed in 11 neoplasms (Supplementary Materials 15 and 16), highlighting the critical role of this pathway in multiple neoplasms. Additionally, *SKP2* was found to be associated with specific signaling pathways (e.g., “complement and coagulation cascades,” “cytokine–cytokine receptor interaction,” and “metabolism of xenobiotics by cytochrome P450”) in at least two cancers (Supplementary Material 16), which required further experimental investigation. At the same time, it is also necessary to further study the molecular mechanism of *SKP2* in specific cancers. For example, *SKP2* may play its roles in GBM and SARC through complex mechanisms (at least five signaling pathways) (Supplementary Materials 17 and 18).

### Importance of ***SKP2*** in a variety of cancer cells and exploration of potential targeted drugs

We further verified the vital role of *SKP2* in various tumors through CRISPR data. The CRISPR results demonstrated the essential cancer role of *SKP2* in a series of neoplasms, including BLCA, CESC, COAD, ESCA, STAD, LIHC, SKCM, PRAD, SARC, THCA, UCS (uterine carcinosarcoma), and UVM (gene effect < 0) (Fig. 9C). In other words, in these neoplasm cells, those with low *SKP2* expression tend to be depleted, suggesting *SKP2* can promote the development of these neoplasms.

**Fig. 10:**
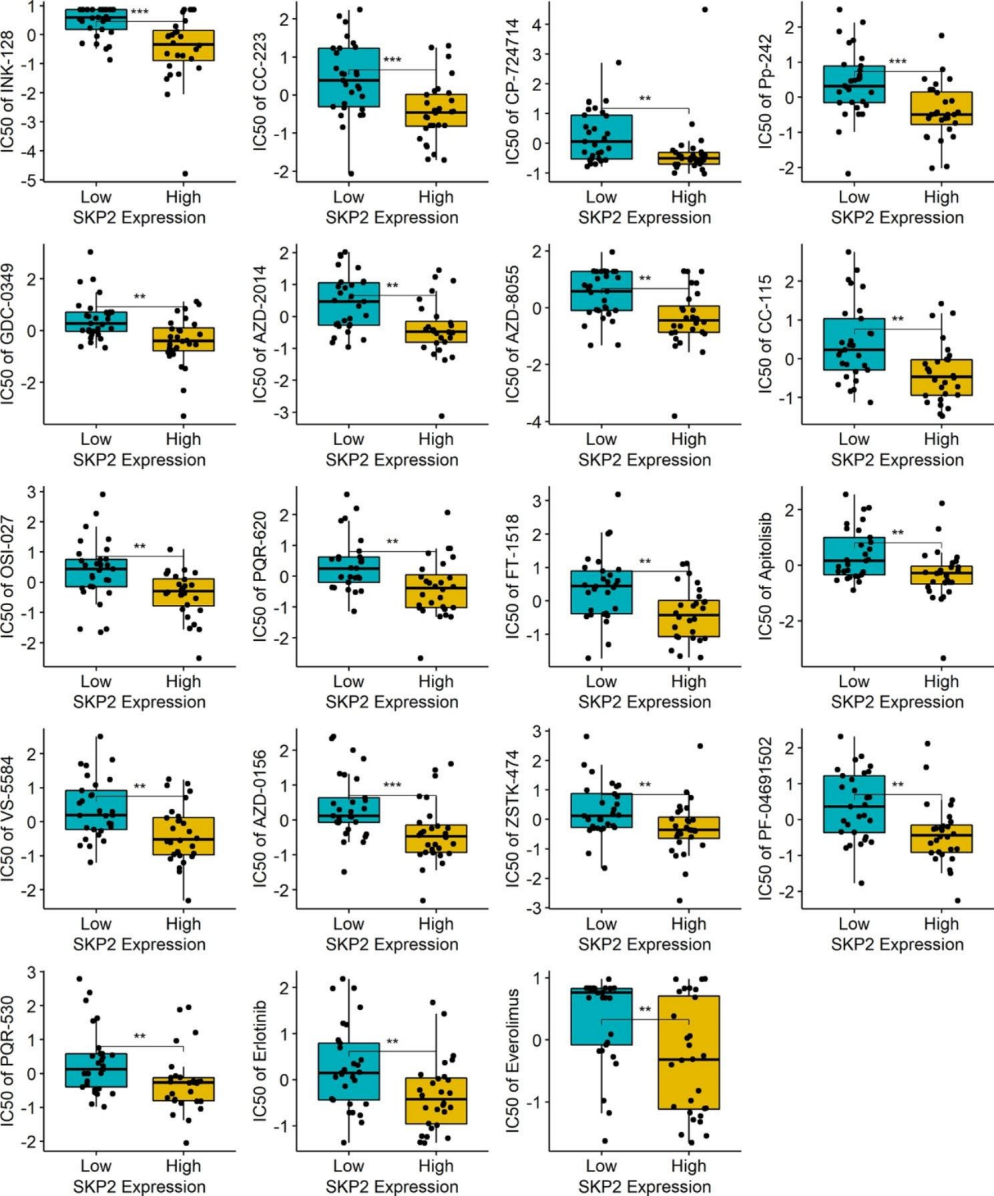
Drug sensitivity of *SKP2*. Drugs with a *p*-value of less than 0.01 are shown in this figure. ^**^p < 0.01; ^***^p < 0.001; *p*-value is based on the Wilcoxon rank-sum test. IC50, half-maximal inhibitory concentration

The above results indicate the importance of *SKP2* in multiple neoplasms, and the gene may be a potential marker for the treatment of several cancers. Thus, a drug sensitivity analysis for *SKP2* was carried out. Through the analysis results based on CellMiner data, 19 types of 57 drugs approved by the American Food and Drug Administration or verified by clinical trials may benefit cancer patients with high-*SKP2* expression, as cancer cells with upregulated *SKP2* expression were susceptible to these drugs based on the results of IC50 (*p* < 0.05) (Fig. 10).

## Discussion

Based on 19,515 samples from several sources, for the first time in an overview of pan-cancer, this study disclosed the overexpression of *SKP2* and its risk factor for the prognosis of patients in multiple neoplasms. The increased expression levels of *SKP2* in tumors may result from regulation of the TF FOXM1. *SKP2* expression made it feasible to distinguish the neoplasm and control tissues of 21 neoplasms, implying its potential in screening a series of neoplasms. Further, the study also investigated the associations between *SKP2* expression and several factors including DNMTs, MMRGs, MSI, TMB, neoantigen count, and immunity, as well as conducted GSEA, and examined drug susceptibility. The study identified the potential of *SKP2* as a marker for the treatment and identification of these neoplasms. It also provided clues to understanding the underlying mechanisms of pan-cancer.

*SKP2* is upregulated in most tumors. Previously, the elevated expression level of *SKP2* was identified in a few cancers, such as COAD [30] and oral squamous cell carcinoma [31]. In our study, distinct *SKP2* expression was found in various normal organ and neoplasm cells. Moreover, *SKP2* mRNA was found to be highly expressed in 15 tumors — BLCA, CESC, CHOL, COAD, ESCA, GBM, HNSCC, KIRC, LIHC, LUAD, LUSC, PCPG, READ, STAD, and UCEC, and the overexpression status of SKP2 in cancer tissues of CESC, COAD, and STAD was verified at the protein level. By contrast, a low expression of *SKP2* mRNA was observed in tissues of KICH, PRAD, and THCA. Taken together, the overexpression of *SKP2* was identified in most neoplasms.

The regulation mechanisms of SKP2 expression in tumors remain unclear. Based on our study, the increased expression levels of *SKP2* in tumors may result from regulation of the TF FOXM1, as evidenced by (1) the high expression of both *FOXM1* and *SKP2* in 15 cancers (CESC, etc.); (2) positive correlations between the expressions of *FOXM1* and *SKP2 *in the 15 tumors; and (3) the presence of binding peaks of FOXM1 upstream of the transcription start site of *SKP2*. However, the possible mechanisms of *SKP2* expression in cancers may be complicated. An example is that a downregulation of *SKP2* expression levels was identified in LAML patients with chromosome 5 alternations compared to those without chromosome 5 alternations. In addition to this, according to previous studies, amplification of the *SKP2* locus was reported in certain cancers, such as in lung cancer, biliary tract cancer, and glioblastoma [32–34], which may contribute to the upregulation of *SKP2* in these cancers at transcriptional levels. However, considering that the number of samples with gene amplification was far smaller than that of samples with high *SKP2* expression in certain cancers (e.g., small-cell lung carcinoma) [35], it is possible that high SKP2 expression cannot be attributed solely to gene amplification. Indeed, abnormal SKP2 expression was also identified in the absence of *SKP2* locus amplification, and regulation at post-transcriptional levels may play a role in this process [36]. Thus, further research is needed to explore the mechanisms of high SKP2 expression in tumors.

High expression of *SKP2* is related to the poor prognosis of tumors. Bochis et al. [30] revealed that high *SKP2* expression predicted higher tumor-node-metastasis stages in patients with COAD and was related to poor OS and relapse-free survival. Yamada et al. [31] identified that over-expressed *SKP2* was a signal of poor prognosis for patients with oral squamous cell carcinoma. They confirmed that downregulation of *SKP2* could significantly reduce the migration and invasion ability of oral squamous cell carcinoma cells. In several tumors, such as breast cancer, osteosarcoma, and glioblastoma, overexpression of *SKP2* was associated with poor prognosis [6, 37, 38]. Based on our pan-cancer study, increased *SKP2* was a risk factor for the prognosis (OS, DSS, DFI, and/or PFI) of individuals with one of ten types of cancers — ACC, KICH, KIRP, LGG, LIHC, MESO, PRAD, SKCM, THCA, and UVM — while it predicted a favorable prognosis in READ, THYM, and OV patients. Thus, the risk role of upregulated *SKP2* expression is detected in most tumors, demonstrating the clinical value of the gene for prognosis. Based on this finding, a potential practical application example is to detect the expression level of *SKP2* in cancer patients by some techniques (e.g., microarray, RNA-sequence or quantitative polymerase chain reaction) in the future, and to predict the prognosis of the patients according to the expression level of *SKP2*, thereby providing some reference for the clinical management of these patients.

A few studies have reported the potential of *SKP2* as a prognostic marker, but its identification effect on cancer status has not been investigated. Based on the AUC analysis of the current study, *SKP2* is a remarkable molecule to distinguish cancer tissues and normal tissues for various cancers (particularly BLCA, CESC, COAD, ESCA, GBM, LUSC, PCPG, and READ). Such a result suggests that *SKP2* is a potential biomarker for predicting cancer status in multiple tumors.

This study preliminarily discussed the underlying mechanisms of *SKP2* in multiple neoplasms by investigating the correlation of *SKP2* with DNMTs, MMRGs, MSI, and TMB. Epigenetic alterations can influence biological processes (e.g., growth and proliferation) of neoplasms. DNMT-mediated DNA methylation and MMRG-led MMR are common epigenetic modifications, enabling DNMTs and MMRGs to impact disease development. For example, *DNMT1*, *DNMT3A*, and *DNMT3B* have been shown to promote LAML, which may be due to DNMTs silencing certain tumor suppressor genes through hypermethylation of these genes, thereby promoting the progression of LAML [25]. Dysfunction of MMRGs may lead to the loss of control of various biological processes, thereby affecting tumor formation and treatment [26]. In our study, the expression level of *SKP2* was significantly positively correlated with the expression levels of three DNMTs and five MMRGs, suggesting that the effect of *SKP2* on DNMT and MMR may be one of the critical pathways for the gene to promote cancer. Dysfunctions of MMR can cause MSI, and elevated levels of MSI exhibit high TMB [39, 40]. Increased MSI and TMB are associated with elevated neoantigen, thereby stimulating or promoting the body’s immune response; thus, MSI and TMB are considered practical markers for predicting response to immunotherapy [41–43]. *SKP2* was not only positively correlated with the MSI and TMB of some cancers (e.g., COAD, READ, and SARC), but also positively associated with immune neoantigen count. This suggests that *SKP2* may also have the potential to predict the immune response to immunotherapy, which requires further investigation.

The relationship between *SKP2* and the immune microenvironment, including immune cell levels, is very complex in many tumors. On the one hand, *SKP2* was positively correlated with six immune cell infiltration levels (B cells, CD4 T cells, CD8 T cells, neutrophil cells, macrophage cells, and dendritic cells) in some tumors (especially THYM, KICH, and KIRC), suggesting that *SKP2* may act as an immune antigen to activate the immune response, thereby causing an increase in immune cell levels. Such a finding has also been supported in some single cancers. For example, Kim et al. [44] found that upregulated *SKP2* was positively correlated with the level of regulatory T cell infiltration. Alvaro et al. [45] reported that Hodgkin lymphoma cells with high *SKP2* expression had higher T cell infiltration levels than those with low *SKP2* expression. On the other hand, *SKP2* was negatively correlated with immune microenvironment scores in some tumors (such as ACC, SKCM, TGCT, and GBM), suggesting that *SKP2* may also be involved in significantly negative regulation of the immune microenvironment in these tumors and then affect the prognosis of patients. However, previous research has not provided similar reference information. Therefore, the current studies suggest that *SKP2* may have two sides in immune regulation for different tumors, but further investigations are necessary.

This study also suggested the potential direction of *SKP2* in tumor-related research and the drugs targeting this molecule. The protein encoded by cyclin dependent kinase inhibitor 1B (*CDKN1B*/p27) is a negative regulator of the cell cycle. The complex composed of this protein enables cells to stay in the G1 phase of their cycle and, thus, it has the effect of inhibiting tumors. As a ubiquitin ligase subunit, SKP2 can target to degrade CDKN1B, promote the transformation of cells from the G1 phase to the S phase, and then cause the growth and proliferation of cancer cells; this is considered the typical function of SKP2 [46–48]. It is noteworthy that, based on the GSEA focusing on a common potential molecular mechanism in different neoplasms, the olfactory transduction pathway may also be essential for SKP2 to affect cancer progression, as the pathway was observed in up to 11 neoplasms. Indeed, olfactory transduction has been found to be related to the occurrence and development of tumors. For example, a series of olfactory receptors are identified as cancer-driven factors [49–51]. However, whether SKP2 affects the process of cancer through an olfactory transmission mechanism requires further experimental verification. In other aspects, the CRISPR data from DepMap Portal confirmed the essential cancer role of *SKP2* in certain tumors (BLCA, etc.), suggesting that *SKP2* promotes cancer in these tumors. Further, the current study revealed a series of drugs that may be suitable for neoplasm patients with high *SKP2* expression.

This study has certain limitations. Due to limited specimens, *SKP2* mRNA expression differences in various cancers were not verified at the protein level, and there was an imbalance in sample size between the experimental and control groups in the TARGET-AML dataset. In addition, no body-fluid samples were collected to detect *SKP2* screening for pan-cancer. More studies are needed to verify the molecular mechanism of SKP2 in various cancers.

Collectively, this study provided a comprehensive analysis of *SKP2* in human neoplasms. *SKP2* plays an essential role in multiple neoplasms and may serve as a marker for treating and identifying these neoplasms.

## Electronic supplementary material

Below is the link to the electronic supplementary material.

**Supplementary Material 1.** THPA samples included in this study for exploring SKP2 protein levels

**Supplementary Material 2.** Age distribution differences between cancer group and normal group

**Supplementary Material 3.** Gender distribution differences between cancer group and normal group

**Supplementary Material 4.** The SKP2 expression trends at protein levels in multiple neoplasms

**Supplementary Material 5.** The potential association between transcription factor FOXM1 and SKP2 in pan-cancer. Panel A: Expression of FOXM1 was significantly upregulated in 15 human cancer types, as determined by Wilcoxon rank-sum test (multiple comparison test by false discovery rate, **p < 0.01, ***p < 0.001). Panel B: The mRNA expression levels of FOXM1 were positively correlated with those of SKP2 in 15 cancer types (Spearman correlation coefficients are indicated as numerical values). Panel C: ChIP-Seq binding peaks of FOXM1 were observed upstream of SKP2 transcriptional start sites

**Supplementary Material 6.** SKP2 expression between LAML patients with chromosome 5 alternations and those without chromosome 5 alternations. A: Wilcoxon rank-sum test based on original data. B: Wilcoxon rank-sum test based on SMOTE data

**Supplementary Material 7.** The relationship of SKP2 expression with cancer patients’ AJCC stage

**Supplementary Material 8.** The relationship of SKP2 expression with cancer patients’ gender

**Supplementary Material 9.** The relationship of SKP2 expression with cancer patients’ age

**Supplementary Material 10.** Identification of covariate between age, gender, and AJCC stage

**Supplementary Material 11.** The relationship of SKP2 expression with KIRP patients’ age and AJCC stage

**Supplementary Material 12.** The associations between SKP2 expression and immune cell infiltration levels

**Supplementary Material 13.** The associations between SKP2 expression and immune environment

**Supplementary Material 14.** The relationship of SKP2 expression with immune checkpoints gene expression

**Supplementary Material 15.** KEGG results based on gene set enrichment analysis in this study

**Supplementary Material 16.** A summary of KEGG results based on gene set enrichment analysis in this study

**Supplementary Material 17.** The potential signaling pathways of SKP2 in GBM

**Supplementary Material 18.** The potential signaling pathways of SKP2 in SARC

## Data Availability

The data supporting pan-cancer analyses’ findings are available in GTEx Portal at https://gtexportal.org/home/, Depmap Portal at https://depmap.org/portal/download/, THPA at https://www.proteinatlas.org/, and the Cancer Genome Atlas at https://www.cancer.gov/about-nci/organization/ccg/research/structural-genomics/tcga.
