# Supplementary material for: Gene S-phase kinase associated protein 2 is a novel prognostic marker in human neoplasms
Source: BMC Med Genomics. 2023 Jun 12;16:128. doi: 10.1186/s12920-023-01561-4 (PMC10259050; doi:10.1186/s12920-023-01561-4)
Supplement: Supplementary file 1 — Supplementary Material 1. THPA samples included in this study for exploring SKP2 protein levels [file 12920_2023_1561_MOESM1_ESM.pdf]

Supplementary Material 1. THPA samples included in this study for exploring SKP2 protein levels.

Patient ID	Gender	Age in year	Cancer	Group	Antibody	Intensity	Quantity	IHC Score
3517	Female	60	BLCA	Tumor	HPA054633	Negative	None	0
3890	Male	41	BLCA	Tumor	HPA054633	Weak	<25%	1
1906	Male	57	BLCA	Tumor	HPA054633	Weak	75%-25%	2
3516	Female	63	BLCA	Tumor	HPA054633	Weak	75%-25%	2
1984	Male	61	BLCA	Tumor	HPA054633	Weak	>75%	3
3111	Female	78	BLCA	Tumor	HPA054633	Weak	>75%	3
3465	Female	56	BLCA	Normal	HPA054633	Moderate	>75%	6
3497	Male	37	BLCA	Normal	HPA054633	Moderate	>75%	6
1760	Male	56	BLCA	Tumor	HPA054633	Moderate	>75%	6
1871	Female	85	BLCA	Tumor	HPA054633	Moderate	>75%	6
2469	Male	78	BLCA	Tumor	HPA054633	Moderate	>75%	6
3079	Female	70	BLCA	Tumor	HPA054633	Moderate	>75%	6
4467	Male	91	BLCA	Tumor	HPA054633	Strong	>75%	9
4719	Female	79	BLCA	Tumor	HPA054633	Strong	>75%	9
2005	Female	55	CESC	Normal	CAB013533	Weak	<25%	1
2434	Female	65	CESC	Normal	CAB013533	Weak	<25%	1
3313	Female	39	CESC	Normal	CAB013533	Weak	<25%	1
1716	Female	39	CESC	Tumor	CAB013533	Moderate	75%-25%	4
1751	Female	34	CESC	Tumor	CAB013533	Moderate	75%-25%	4
2480	Female	44	CESC	Tumor	CAB013533	Moderate	<25%	2
2528	Female	44	CESC	Tumor	CAB013533	Moderate	>75%	6
2841	Female	32	CESC	Tumor	CAB013533	Moderate	75%-25%	4
2988	Female	38	CESC	Tumor	CAB013533	Moderate	75%-25%	4
3382	Female	70	CESC	Tumor	CAB013533	Moderate	75%-25%	4
3400	Female	46	CESC	Tumor	CAB013533	Moderate	<25%	2
3409	Female	63	CESC	Tumor	CAB013533	Moderate	>75%	6
1958	Female	84	COAD	Normal	HPA054633	Weak	75%-25%	2
1960	Male	84	COAD	Normal	HPA054633	Weak	75%-25%	2
2931	Male	77	COAD	Tumor	HPA054633	Weak	75%-25%	2
1898	Male	71	COAD	Tumor	HPA054633	Moderate	75%-25%	4
2616	Female	67	COAD	Tumor	HPA054633	Moderate	75%-25%	4
4089	Female	67	COAD	Tumor	HPA054633	Moderate	75%-25%	4
4094	Female	86	COAD	Tumor	HPA054633	Moderate	>75%	6
4382	Female	43	COAD	Tumor	HPA054633	Moderate	>75%	6
4453	Female	78	COAD	Tumor	HPA054633	Moderate	>75%	6
4606	Female	48	COAD	Tumor	HPA054633	Moderate	>75%	6
4721	Female	84	COAD	Tumor	HPA054633	Moderate	>75%	6
4745	Male	62	COAD	Tumor	HPA054633	Moderate	>75%	6
3722	Female	80	HNSCC	Tumor	CAB013491	Negative	None	0
4424	Female	58	HNSCC	Normal	CAB013491	Negative	None	0

3917	Female	70	HNSCC	Normal	CAB013491	Negative	None	0
3406	Male	54	HNSCC	Normal	CAB013491	Negative	None	0
4116	Male	52	HNSCC	Tumor	CAB013491	Strong	<25%	3
4117	Male	69	HNSCC	Tumor	CAB013491	Strong	75%-25%	6
1859	Male	61	Kidney	Normal	CAB013491	Negative	None	0
2530	Female	41	Kidney	Normal	CAB013491	Negative	None	0
3229	Male	59	Kidney	Normal	CAB013491	Negative	None	0
1831	Male	77	Kidney	Tumor	CAB013491	Negative	None	0
1901	Female	69	Kidney	Tumor	CAB013491	Negative	None	0
1969	Male	63	Kidney	Tumor	CAB013491	Negative	None	0
2210	Female	64	Kidney	Tumor	CAB013491	Negative	None	0
2477	Male	80	Kidney	Tumor	CAB013491	Negative	None	0
3039	Female	67	Kidney	Tumor	CAB013491	Negative	None	0
3541	Female	59	Kidney	Tumor	CAB013491	Negative	None	0
3616	Female	63	Kidney	Tumor	CAB013491	Negative	None	0
3156	Male	63	Kidney	Tumor	CAB013491	Weak	<25%	1
848	Male	58	Kidney	Tumor	CAB013491	Moderate	<25%	2
1113	Female	59	Kidney	Tumor	CAB013491	Moderate	<25%	2
2261	Male	59	Kidney	Tumor	CAB013491	Moderate	<25%	2
1222	Female	43	LIHC	Tumor	HPA054633	Negative	None	0
2766	Female	73	LIHC	Tumor	HPA054633	Negative	None	0
3196	Male	65	LIHC	Tumor	HPA054633	Negative	None	0
3324	Male	70	LIHC	Tumor	HPA054633	Negative	None	0
879	Male	70	LIHC	Tumor	HPA054633	Weak	<25%	1
3477	Male	67	LIHC	Tumor	HPA054633	Weak	<25%	1
4791	Female	82	LIHC	Tumor	HPA054633	Weak	<25%	1
1720	Male	67	LIHC	Normal	HPA054633	Weak	75%-25%	2
3222	Female	63	LIHC	Normal	HPA054633	Weak	75%-25%	2
3402	Female	54	LIHC	Normal	HPA054633	Weak	75%-25%	2
2325	Male	76	LIHC	Tumor	HPA054633	Moderate	>75%	6
1327	Male	64	LUAD	Tumor	HPA054633	Negative	None	0
4885	Female	51	LUAD	Tumor	HPA054633	Negative	None	0
1470	Male	65	LUAD	Normal	HPA054633	Weak	<25%	1
1678	Female	57	LUAD	Normal	HPA054633	Weak	<25%	1
2208	Female	67	LUAD	Normal	HPA054633	Weak	<25%	1
4867	Female	57	LUAD	Tumor	HPA054633	Weak	75%-25%	2
4866	Female	54	LUAD	Tumor	HPA054633	Moderate	75%-25%	4
4888	Female	52	LUAD	Tumor	HPA054633	Moderate	75%-25%	4
1847	Male	64	LUAD	Tumor	HPA054633	Moderate	>75%	6
1470	Male	65	LUSC	Normal	HPA054633	Weak	<25%	1
1678	Female	57	LUSC	Normal	HPA054633	Weak	<25%	1
2208	Female	67	LUSC	Normal	HPA054633	Weak	<25%	1
1048	Male	68	LUSC	Tumor	HPA054633	Weak	<25%	1
4857	Male	67	LUSC	Tumor	HPA054633	Weak	<25%	1

4860	Male	79	LUSC	Tumor	HPA054633	Moderate	<25%	2
4900	Female	61	LUSC	Tumor	HPA054633	Moderate	<25%	2
4366	Male	68	PRAD	Tumor	HPA054633	Negative	None	0
4364	Male	58	PRAD	Tumor	HPA054633	Weak	<25%	1
4341	Male	67	PRAD	Tumor	HPA054633	Weak	75%-25%	2
4347	Male	63	PRAD	Tumor	HPA054633	Moderate	<25%	2
3984	Male	68	PRAD	Tumor	HPA054633	Weak	>75%	3
1798	Male	55	PRAD	Normal	HPA054633	Moderate	75%-25%	4
1984	Male	61	PRAD	Normal	HPA054633	Moderate	75%-25%	4
3497	Male	37	PRAD	Normal	HPA054633	Moderate	75%-25%	4
3454	Male	57	PRAD	Tumor	HPA054633	Moderate	75%-25%	4
4435	Male	61	PRAD	Tumor	HPA054633	Moderate	75%-25%	4
3456	Male	64	PRAD	Tumor	HPA054633	Moderate	>75%	6
3952	Male	65	PRAD	Tumor	HPA054633	Moderate	>75%	6
4326	Male	56	PRAD	Tumor	HPA054633	Moderate	>75%	6
4327	Male	68	PRAD	Tumor	HPA054633	Moderate	>75%	6
4521	Male	62	PRAD	Tumor	HPA054633	Moderate	>75%	6
1650	Male	55	STAD	Normal	HPA054633	Moderate	<25%	2
3044	Male	48	STAD	Normal	HPA054633	Moderate	<25%	2
4847	Male	70	STAD	Normal	HPA054633	Moderate	<25%	2
2326	Female	65	STAD	Tumor	HPA054633	Weak	75%-25%	2
1994	Female	64	STAD	Tumor	HPA054633	Weak	>75%	3
2142	Male	62	STAD	Tumor	HPA054633	Weak	>75%	3
2473	Male	59	STAD	Tumor	HPA054633	Weak	>75%	3
2574	Female	81	STAD	Tumor	HPA054633	Moderate	75%-25%	4
629	Male	71	STAD	Tumor	HPA054633	Moderate	>75%	6
2105	Male	62	STAD	Tumor	HPA054633	Moderate	>75%	6
2378	Male	59	STAD	Tumor	HPA054633	Moderate	>75%	6
2626	Female	79	STAD	Tumor	HPA054633	Moderate	>75%	6
3044	Male	48	STAD	Tumor	HPA054633	Moderate	>75%	6
3492	Male	55	STAD	Tumor	HPA054633	Moderate	>75%	6
3055	Male	77	STAD	Tumor	HPA054633	Strong	>75%	9
2623	Male	77	THCA	Tumor	CAB013533	Weak	<25%	1
3107	Male	75	THCA	Tumor	CAB013533	Weak	<25%	1
3267	Male	33	THCA	Tumor	CAB013533	Weak	<25%	1
3131	Female	30	THCA	Tumor	CAB013533	Moderate	<25%	2
2072	Male	61	THCA	Normal	CAB013533	Moderate	>75%	6
3005	Female	44	THCA	Normal	CAB013533	Moderate	>75%	6
2113	Female	67	UCEC	Tumor	HPA054633	Negative	None	0
1118	Female	61	UCEC	Tumor	HPA054633	Weak	<25%	1
1792	Female	41	UCEC	Normal	HPA054633	Moderate	<25%	2
2941	Female	33	UCEC	Normal	HPA054633	Moderate	<25%	2
4876	Female	25	UCEC	Normal	HPA054633	Moderate	<25%	2
2303	Female	68	UCEC	Tumor	HPA054633	Weak	75%-25%	2

2339	Female	79	UCEC	Tumor	HPA054633	Weak	75%-25%	2
4804	Female	82	UCEC	Tumor	HPA054633	Weak	75%-25%	2
2981	Female	62	UCEC	Tumor	HPA054633	Moderate	<25%	2
4907	Female	65	UCEC	Tumor	HPA054633	Weak	>75%	3
4205	Female	65	UCEC	Tumor	HPA054633	Moderate	75%-25%	4
2971	Male	76	UCEC	Tumor	HPA054633	Moderate	75%-25%	4
621	Female	65	UCEC	Tumor	HPA054633	Moderate	>75%	6
2607	Female	81	UCEC	Tumor	HPA054633	Moderate	>75%	6
3481	Female	51	UCEC	Tumor	HPA054633	Moderate	>75%	6

Notes: Data in this table are available from v21.0.proteinatlas.org.
