# Supplementary material for: Gene S-phase kinase associated protein 2 is a novel prognostic marker in human neoplasms
Source: BMC Med Genomics. 2023 Jun 12;16:128. doi: 10.1186/s12920-023-01561-4 (PMC10259050; doi:10.1186/s12920-023-01561-4)
Supplement: Supplementary file 2 — Supplementary Material 2. Age distribution differences between cancer group and normal group [file 12920_2023_1561_MOESM2_ESM.pdf]

Supplementary Material 2. Age distribution differences between cancer group and normal group.

Cancer	Group	Age (year)		Chi-square test	
		<65	≥65	χ^2	*p* value
BLCA	Normal	5	14	0.871	0.351
	Tumor	150	257		
BRCA	Normal	82	31	0.744	0.388
	Tumor	748	342		
CESC	Normal	2	1	0.123	0.343
	Tumor	265	39		
CHOL	Normal	1	8	2.552	0.110
	Tumor	17	19		
COAD	Normal	11	30	4.205	**0.040***
	Tumor	125	161		
ESCA	Normal	5	8	1.778	0.182
	Tumor	104	77		
GBM	Normal	0	0	/	1.000
	Tumor	96	95		
HNSCC	Normal	24	20	1.252	0.263
	Tumor	326	191		
KICH	Normal	18	7	0.275	0.600
	Tumor	51	15		
KIRC	Normal	43	32	0.676	0.411
	Tumor	330	200		
KIRP	Normal	16	16	1.277	0.258
	Tumor	172	113		
LIHC	Normal	20	30	7.046	**0.008***
	Tumor	220	148		
LUAD	Normal	27	32	0.032	0.858
	Tumor	220	274		
LUSC	Normal	16	34	0.132	0.716
	Tumor	169	320		
PAAD	Normal	3	1	0.410	0.522
	Tumor	82	96		
PCPG	Normal	3	0	/	1.000
	Tumor	153	24		
PRAD	Normal	34	18	0.025	0.875
	Tumor	329	166		
READ	Normal	5	5	0.000	1.000
	Tumor	47	44		
STAD	Normal	13	23	0.521	0.471
	Tumor	173	236		
THCA	Normal	49	10	0.143	0.706
	Tumor	428	76		

UCEC	Normal	10	3	4.206	**0.040***
	Tumor	84	93		

Notes: $^{*}p < 0.05$.
