# Supplementary material for: Gene S-phase kinase associated protein 2 is a novel prognostic marker in human neoplasms
Source: BMC Med Genomics. 2023 Jun 12;16:128. doi: 10.1186/s12920-023-01561-4 (PMC10259050; doi:10.1186/s12920-023-01561-4)
Supplement: Supplementary file 3 — Supplementary Material 3. Gender distribution differences between cancer group and normal group [file 12920_2023_1561_MOESM3_ESM.pdf]

Supplementary Material 3. Gender distribution differences between cancer group and normal group.

Cancer	Group	Gender		Chi-square test	
		Female	Male	χ^2	*p* value
BLCA	Normal	9	10	4.188	**0.041***
	Tumor	106	301		
BRCA	Normal	112	1	0.000	1.000
	Tumor	1079	12		
CESC	Normal	3	0	/	/
	Tumor	304	0		
CHOL	Normal	3	6	0.673	0.412
	Tumor	20	16		
COAD	Normal	21	20	0.480	0.489
	Tumor	130	156		
ESCA	Normal	5	8	3.604	0.058
	Tumor	26	155		
GBM	Normal	0	0	/	1.000
	Tumor	54	98		
HNSCC	Normal	14	30	0.642	0.423
	Tumor	136	382		
KICH	Normal	12	13	0.372	0.542
	Tumor	27	39		
KIRC	Normal	23	52	0.570	0.450
	Tumor	186	344		
KIRP	Normal	10	22	0.401	0.527
	Tumor	75	213		
LIHC	Normal	22	28	2.590	0.108
	Tumor	120	249		
LUAD	Normal	34	25	0.312	0.576
	Tumor	276	237		
LUSC	Normal	14	36	0.104	0.748
	Tumor	129	369		
PAAD	Normal	2	2	0.000	1.000
	Tumor	80	98		
PCPG	Normal	1	2	0.046	0.830
	Tumor	100	77		
PRAD	Normal	0	52	/	/
	Tumor	0	495		
READ	Normal	7	3	1.208	0.272
	Tumor	42	49		
STAD	Normal	13	23	0.010	0.919
	Tumor	146	268		
THCA	Normal	42	17	0.089	0.765
	Tumor	368	136		

UCEC	Normal	13	0	/	/
	Tumor	180	0		

Notes: $^{*}p < 0.05$.
