# Supplementary figures and images for: Gene S-phase kinase associated protein 2 is a novel prognostic marker in human neoplasms
Source: BMC Med Genomics. 2023 Jun 12;16:128. doi: 10.1186/s12920-023-01561-4 (PMC10259050; doi:10.1186/s12920-023-01561-4)

BLCA\_HPA054633

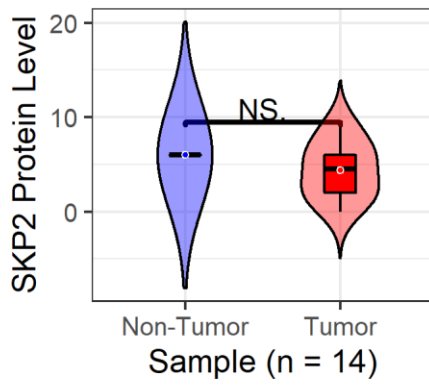

HNSCC\_CAB013491

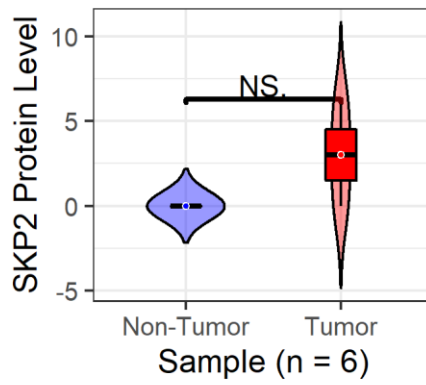

Kidney\_CAB013491

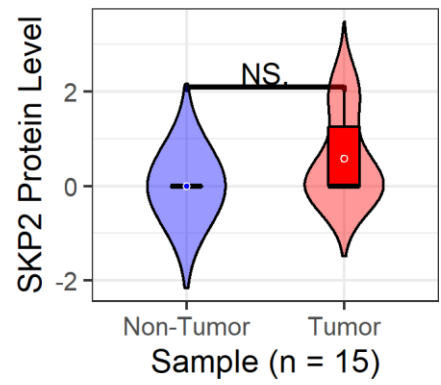

LIHC\_HPA054633

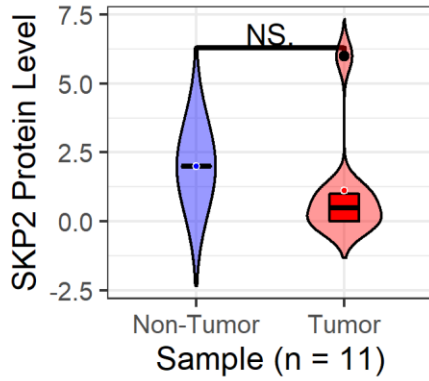

LUAD\_HPA054633

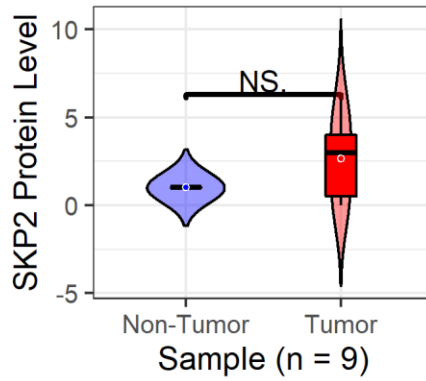

LUSC\_HPA054633

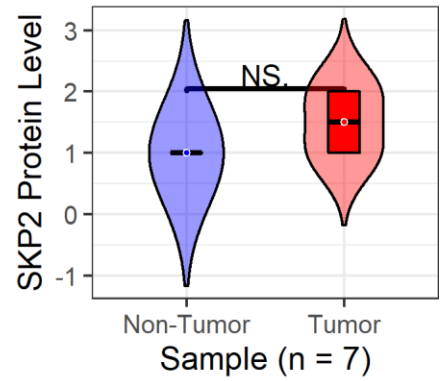

PRAD\_HPA054633

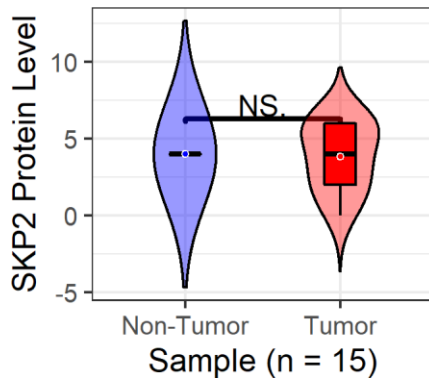

THCA\_CAB013533

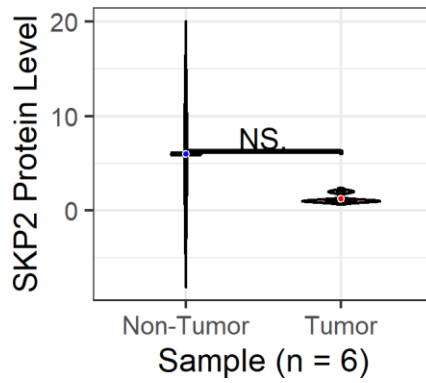

UCEC\_HPA054633

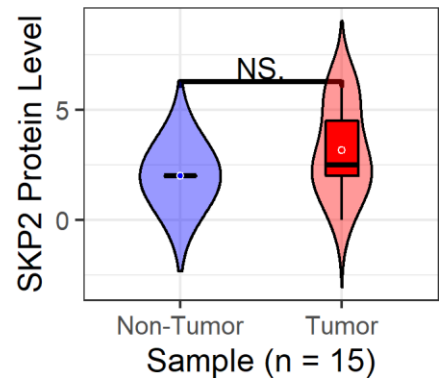

Supplement: Supplementary file 4 — Supplementary Material 4. The SKP2 expression trends at protein levels in multiple neoplasms [file 12920_2023_1561_MOESM4_ESM.pdf]

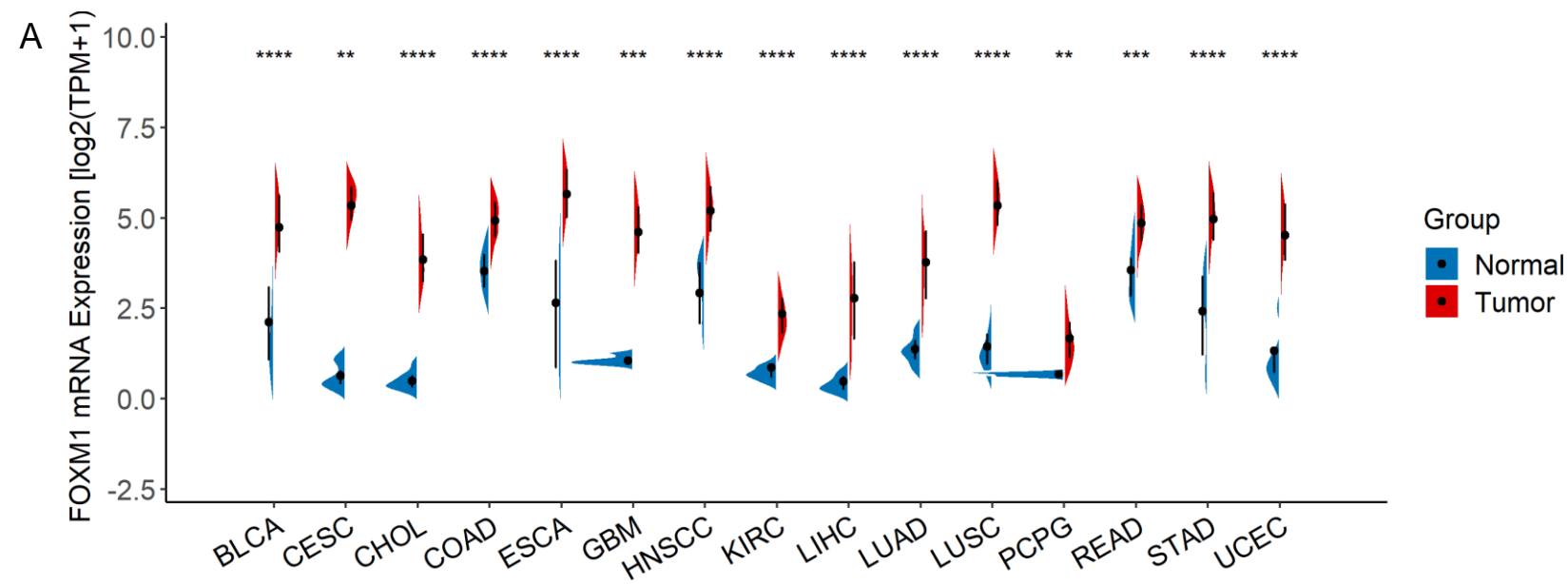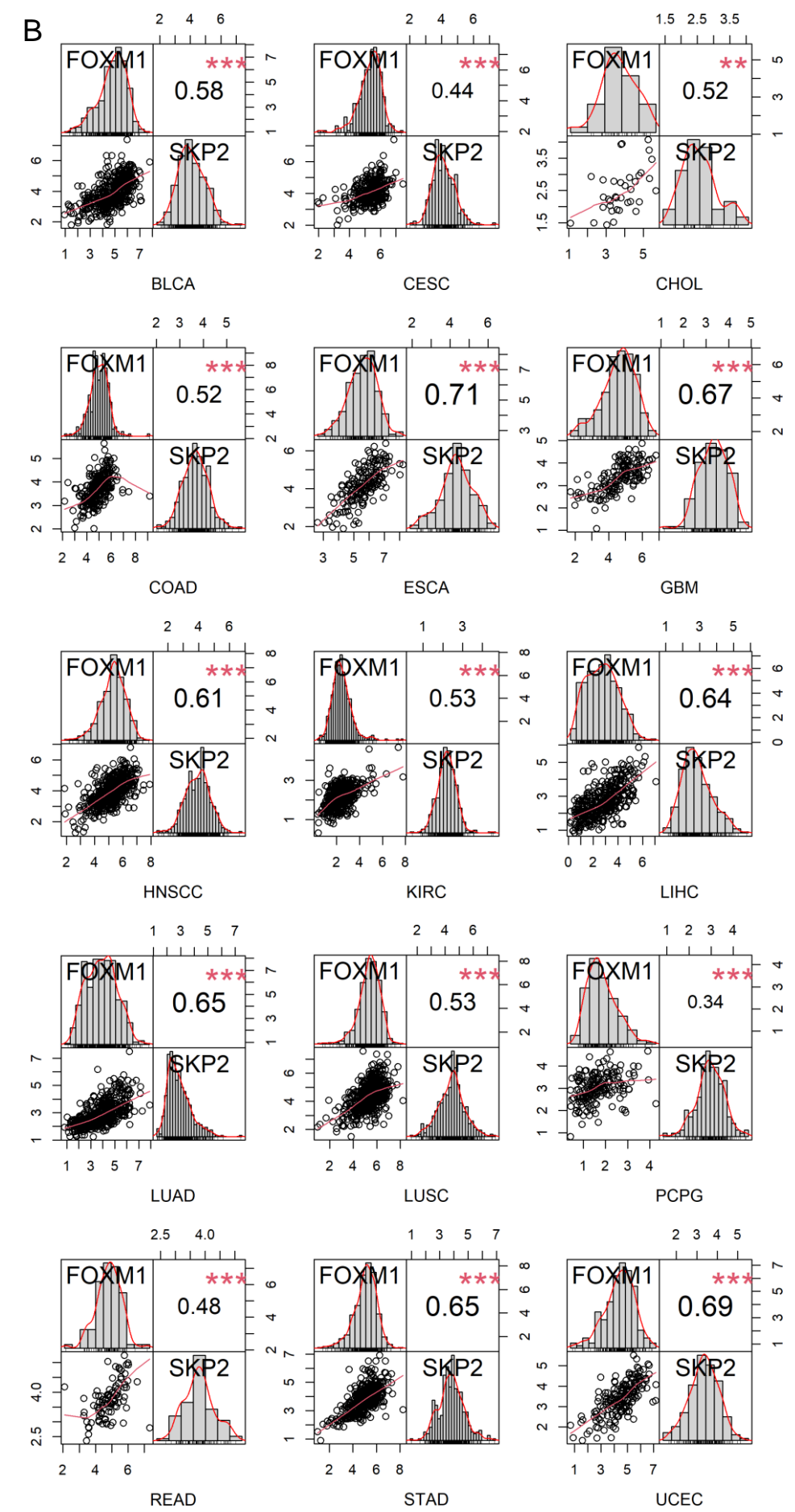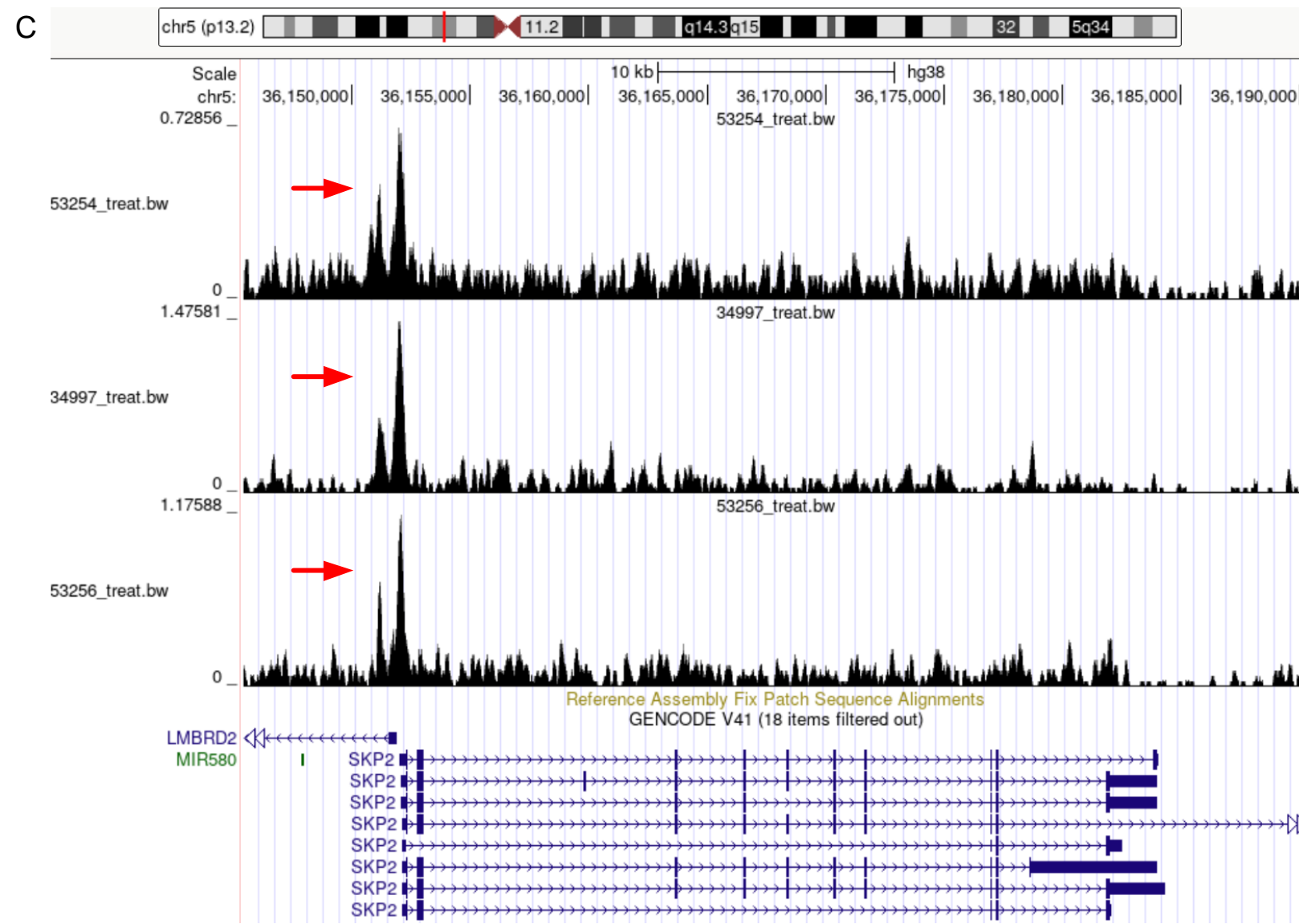

Supplement: Supplementary file 5 — Supplementary Material 5. The potential association between transcription factor FOXM1 and SKP2 in pan-cancer. Panel A: Expression of FOXM1 was significantly upregulated in 15 human cancer types, as determined by Wilcoxon rank-sum test (multiple comparison test by false discovery rate, **p < 0.01, ***p < 0.001). Panel B: The mRNA expression levels of FOXM1 were positively correlated with those of SKP2 in 15 cancer types (Spearman correlation coefficients are indicated as numerical values). Panel C: ChIP-Seq binding peaks of FOXM1 were observed upstream of SKP2 transcriptional start sites [file 12920_2023_1561_MOESM5_ESM.pdf]

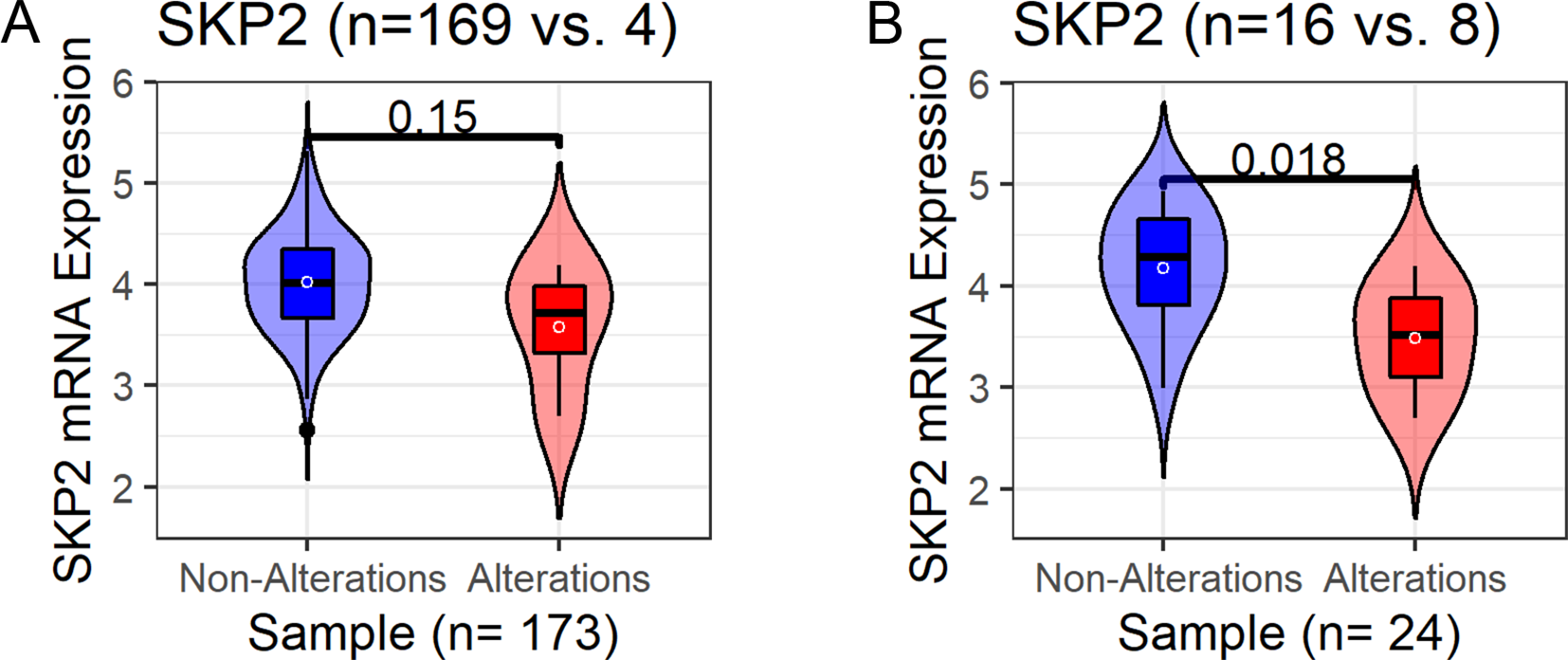

Supplement: Supplementary file 6 — Supplementary Material 6. SKP2 expression between LAML patients with chromosome 5 alternations and those without chromosome 5 alternations. A: Wilcoxon rank-sum test based on original data. B: Wilcoxon rank-sum test based on SMOTE data [file 12920_2023_1561_MOESM6_ESM.tif]

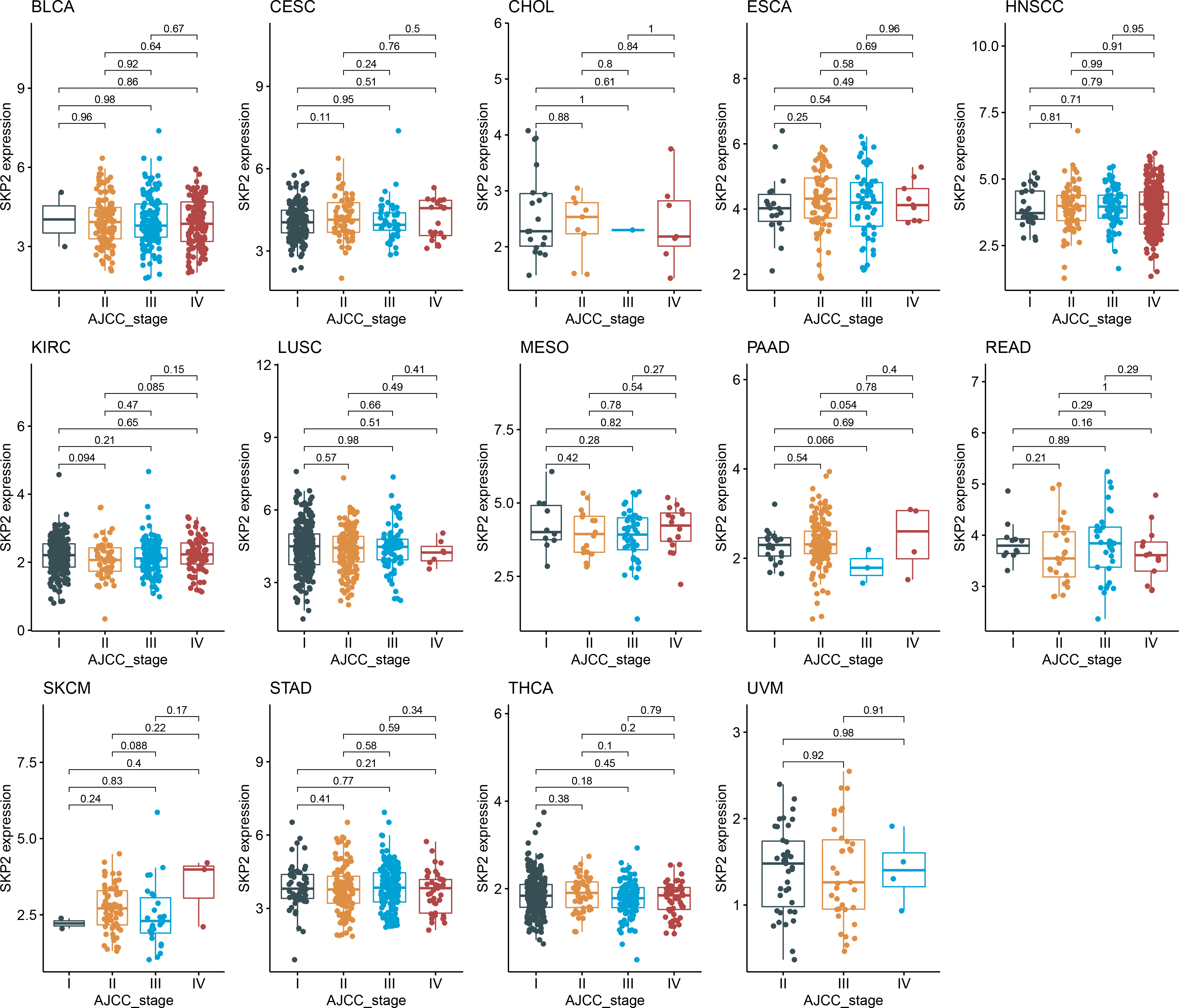

Supplement: Supplementary file 7 — Supplementary Material 7. The relationship of SKP2 expression with cancer patients’ AJCC stage [file 12920_2023_1561_MOESM7_ESM.tif]

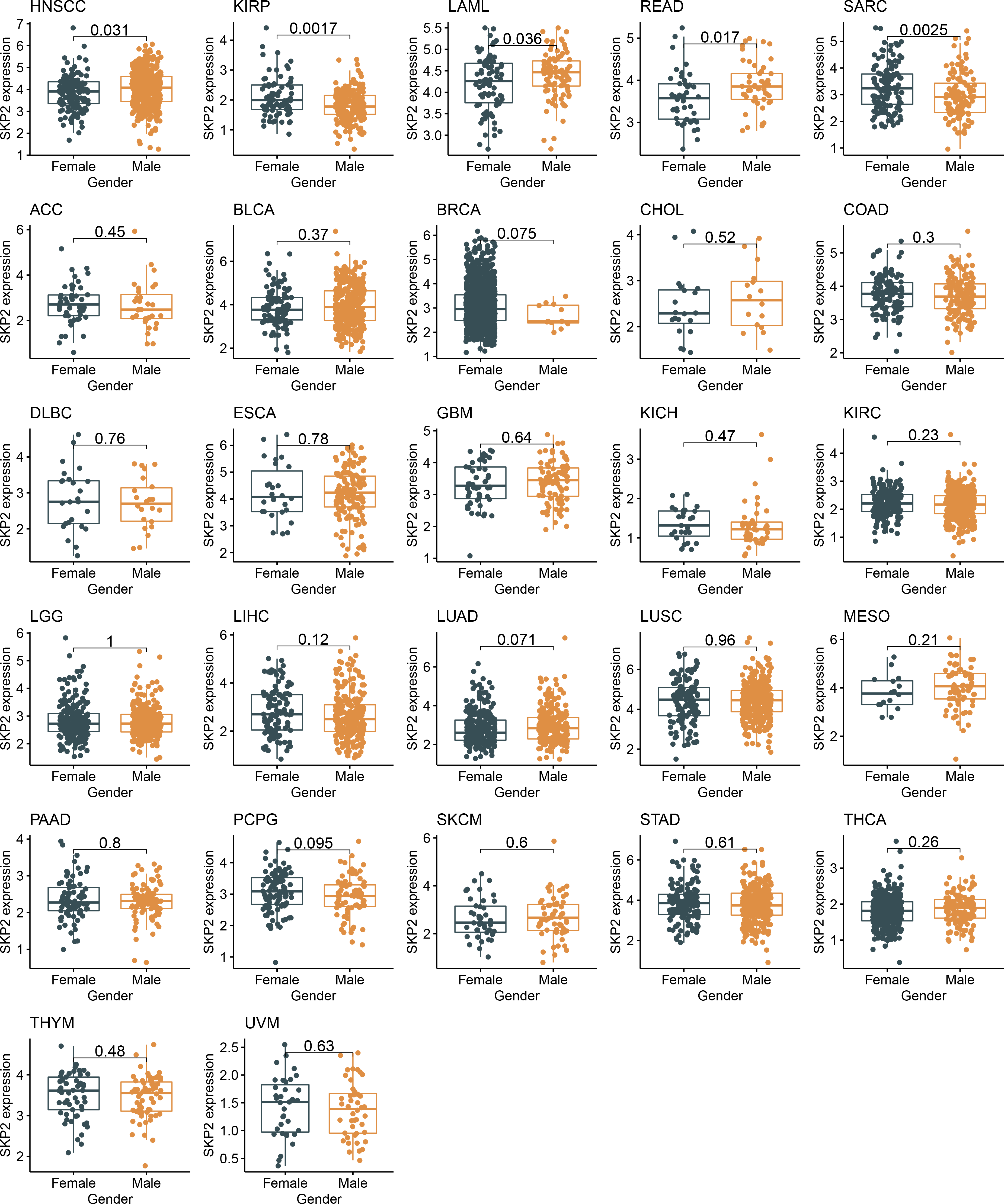

Supplement: Supplementary file 8 — Supplementary Material 8. The relationship of SKP2 expression with cancer patients’ gender [file 12920_2023_1561_MOESM8_ESM.tif]

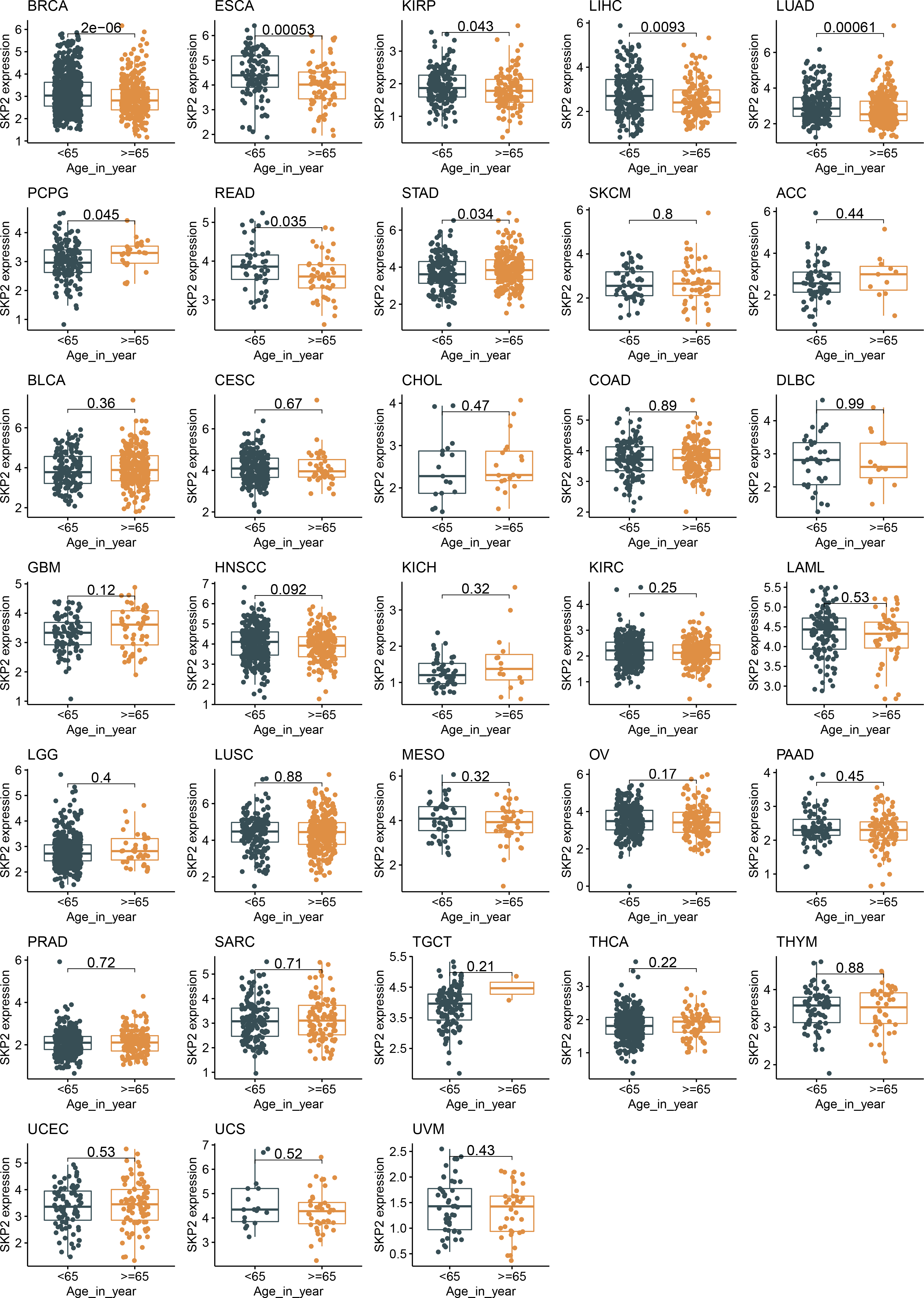

Supplement: Supplementary file 9 — Supplementary Material 9. The relationship of SKP2 expression with cancer patients’ age [file 12920_2023_1561_MOESM9_ESM.tif]

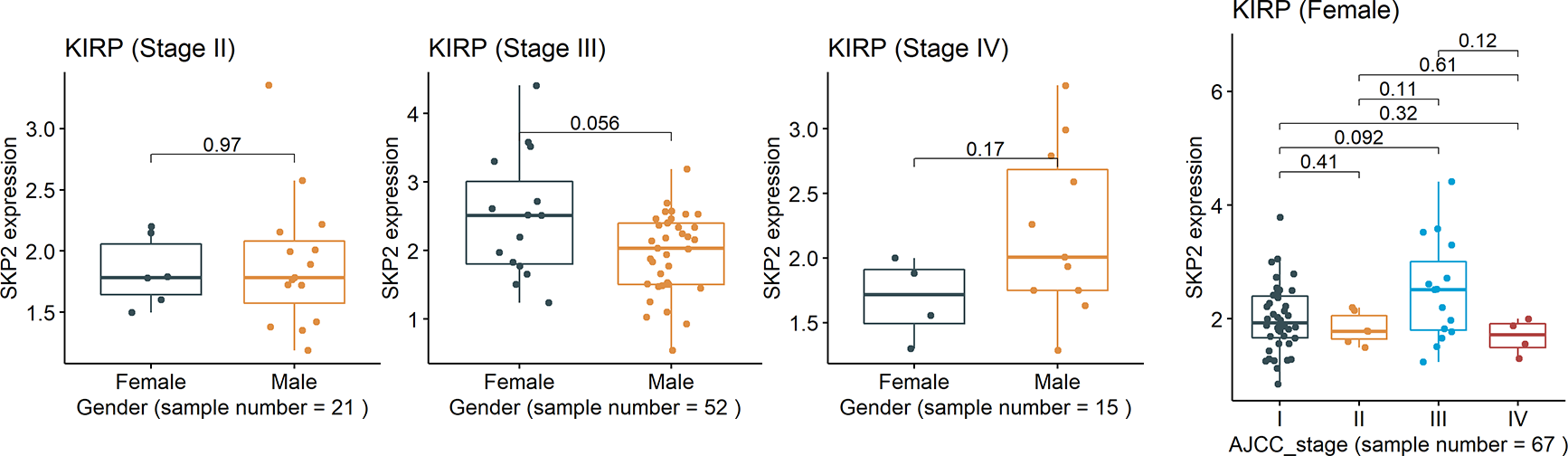

Supplement: Supplementary file 11 — Supplementary Material 11. The relationship of SKP2 expression with KIRP patients’ age and AJCC stage [file 12920_2023_1561_MOESM11_ESM.tif]

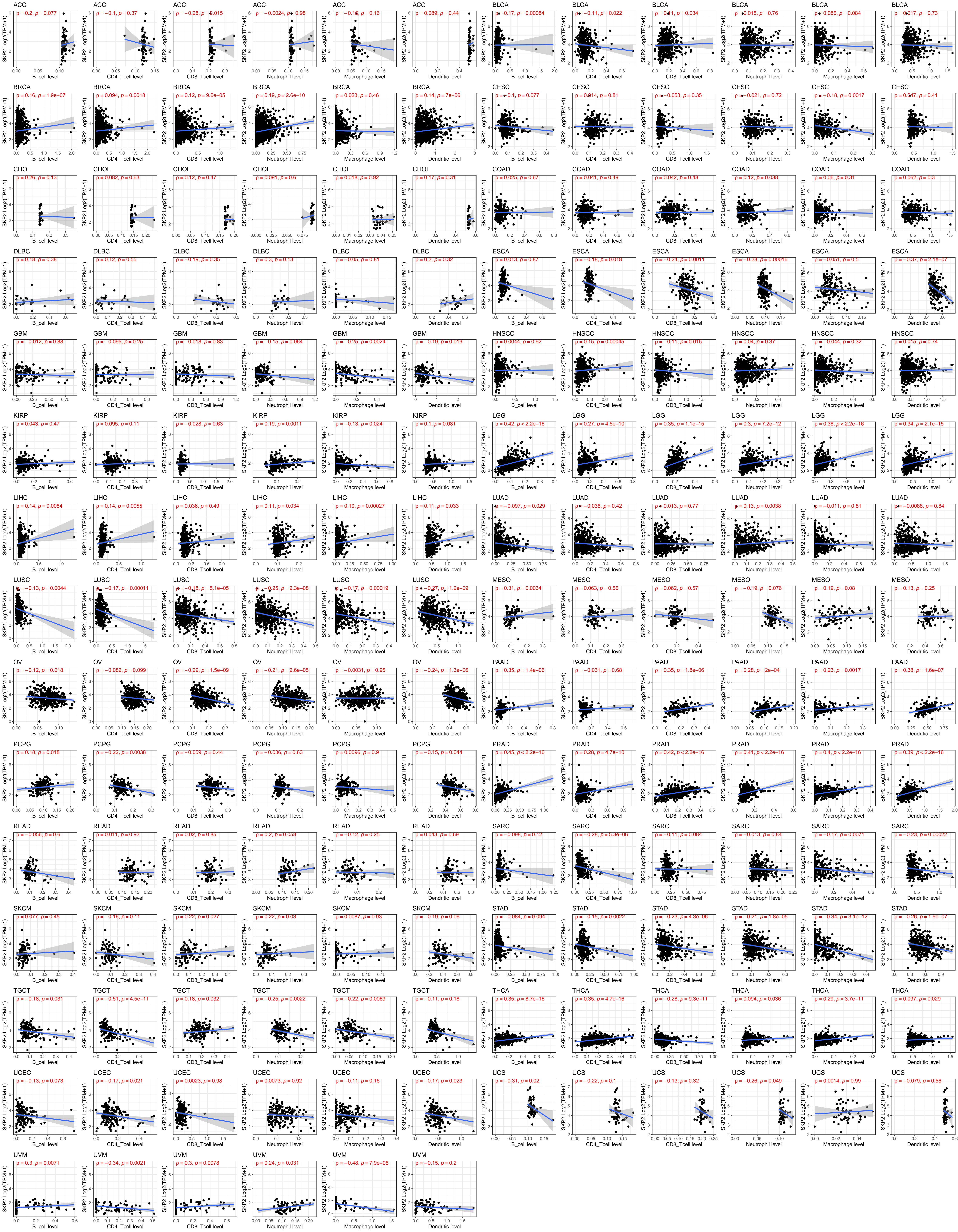

Supplement: Supplementary file 12 — Supplementary Material 12. The associations between SKP2 expression and immune cell infiltration levels [file 12920_2023_1561_MOESM12_ESM.pdf]

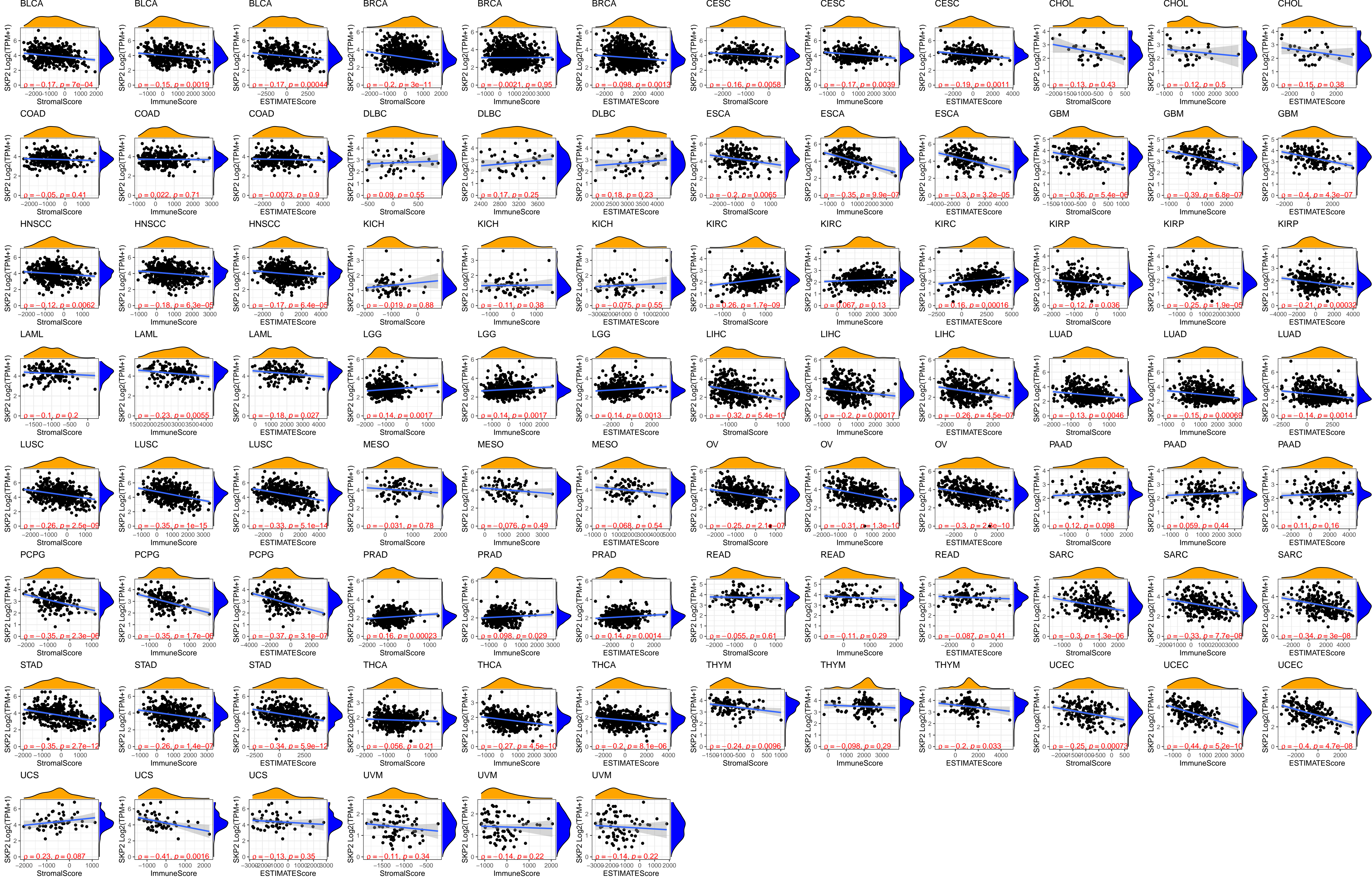

Supplement: Supplementary file 13 — Supplementary Material 13. The associations between SKP2 expression and immune environment [file 12920_2023_1561_MOESM13_ESM.pdf]

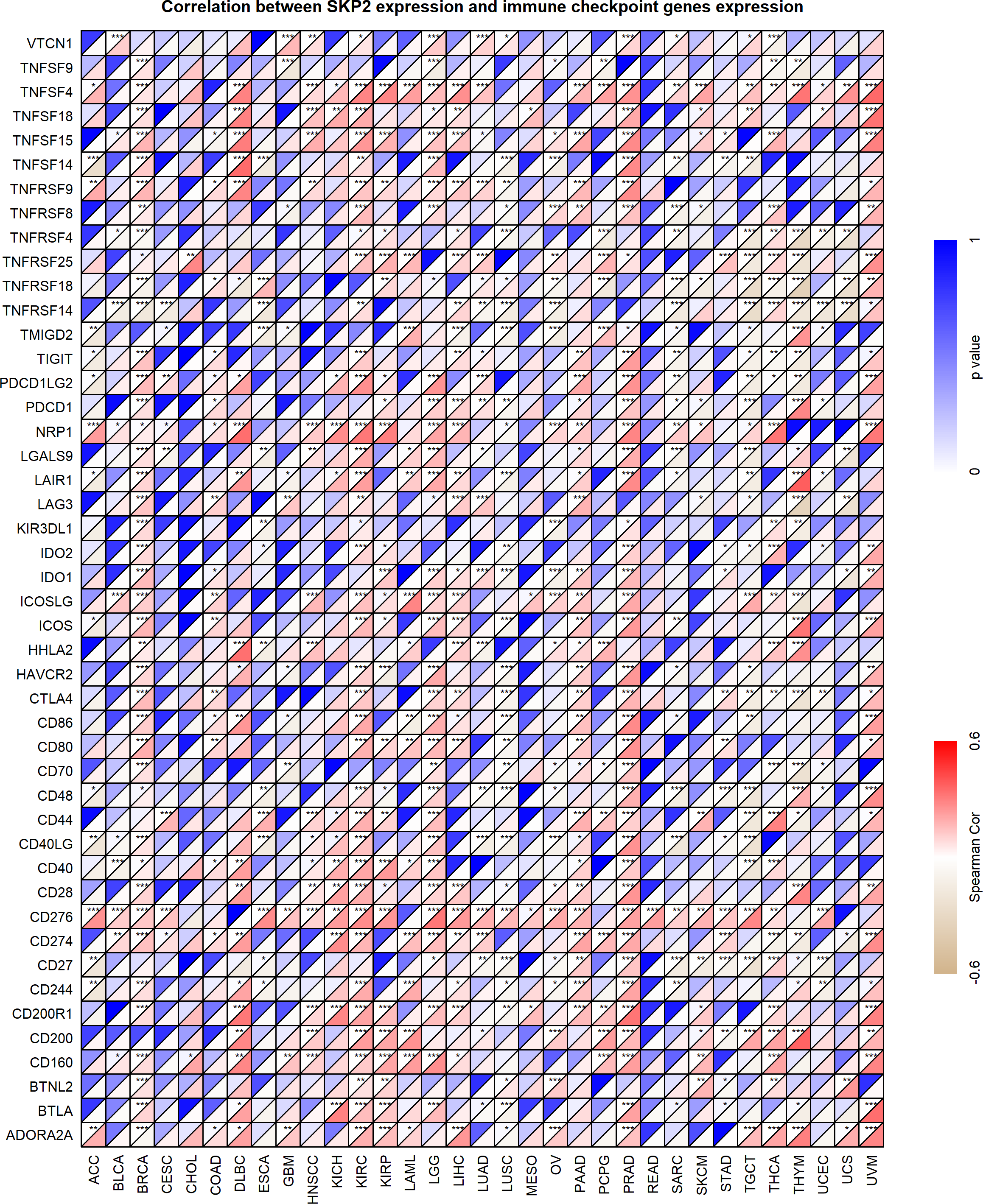

Supplement: Supplementary file 14 — Supplementary Material 14. The relationship of SKP2 expression with immune checkpoints gene expression [file 12920_2023_1561_MOESM14_ESM.tiff]

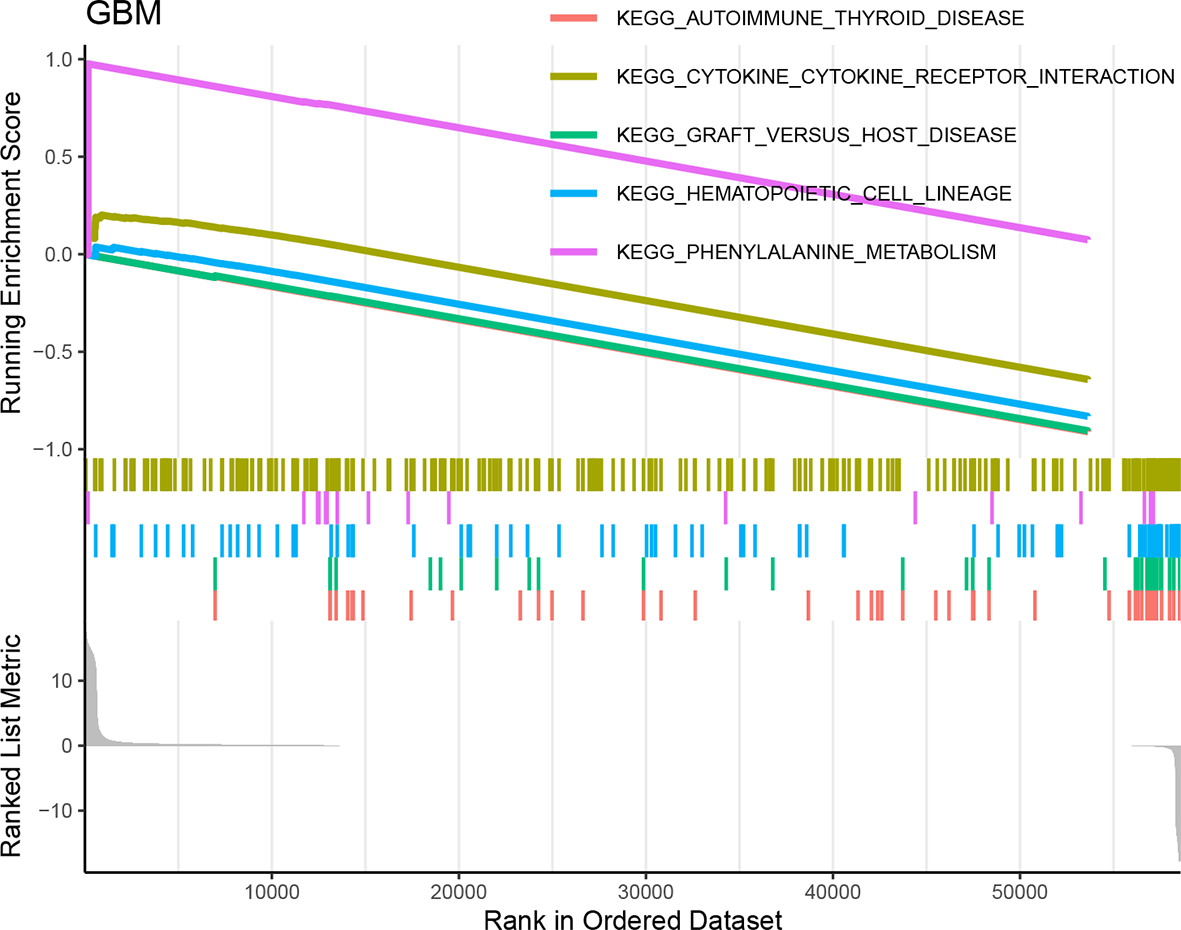

Supplement: Supplementary file 17 — Supplementary Material 17. The potential signaling pathways of SKP2 in GBM [file 12920_2023_1561_MOESM17_ESM.tif]

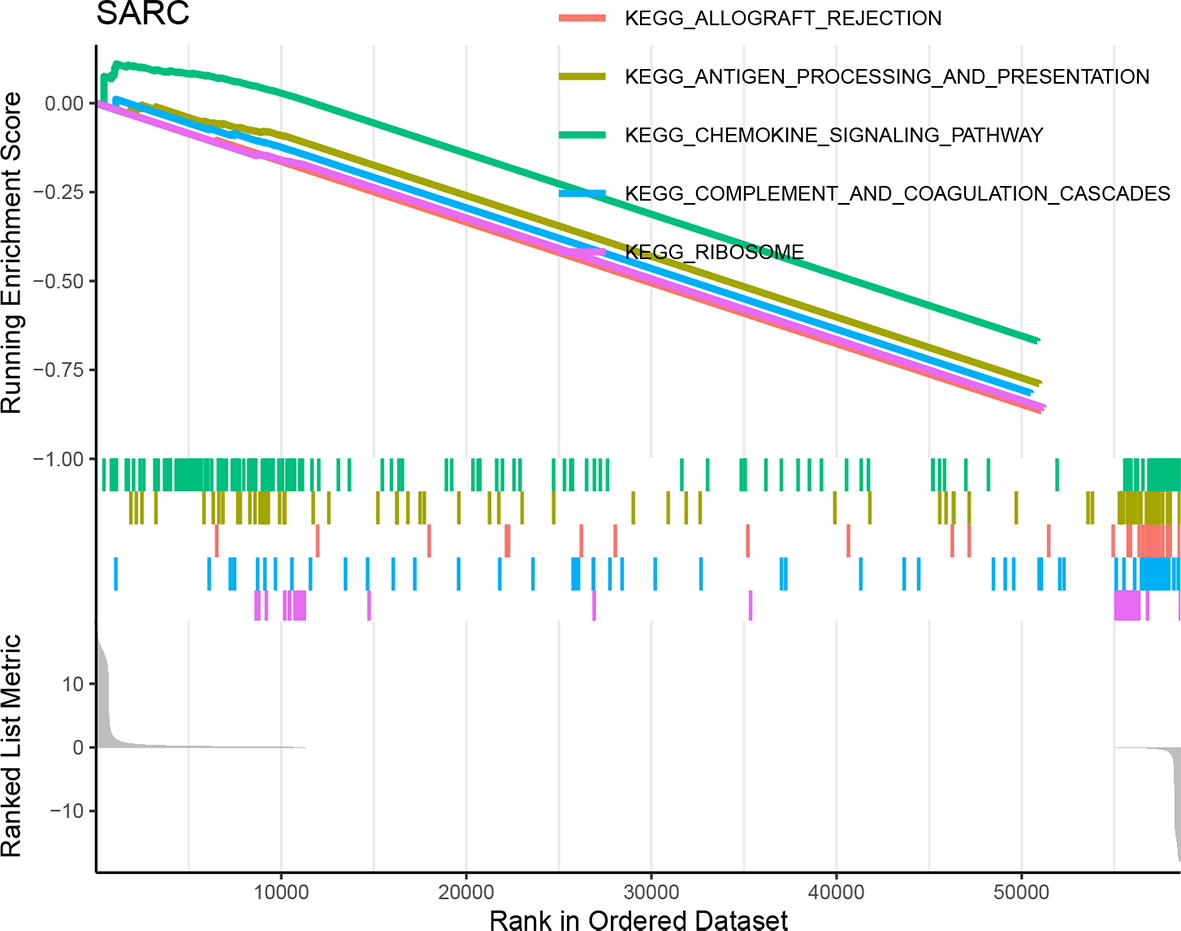

Supplement: Supplementary file 18 — Supplementary Material 18. The potential signaling pathways of SKP2 in SARC [file 12920_2023_1561_MOESM18_ESM.tif]
