# Supplementary material for: Gene S-phase kinase associated protein 2 is a novel prognostic marker in human neoplasms
Source: BMC Med Genomics. 2023 Jun 12;16:128. doi: 10.1186/s12920-023-01561-4 (PMC10259050; doi:10.1186/s12920-023-01561-4)
Supplement: Supplementary file 10 — Supplementary Material 10. Identification of covariate between age, gender, and AJCC stage [file 12920_2023_1561_MOESM10_ESM.pdf]

Supplementary Material 10. Identification of covariate between age, gender, and AJCC stage.

Cancer	Group	Age (years)		Chi-square test		Gender		Chi-square test	
		< 65	⩾ 65	χ^2	*p* value	Female	Male	χ^2	*p* value
BRCA	AJCC I	133	68	2.833	0.418	-	-		
	AJCC II	476	206			-	-		
	AJCC III	185	88			-	-		
	AJCC IV	18	4			-	-		
KIRP	AJCC I	105	79	8.491	**0.037**[*]	47	138	1.150	0.765
	AJCC II	11	10			6	16		
	AJCC III	34	29			20	44		
	AJCC IV	17	2			4	15		
LIHC	AJCC I	105	82	5.334	0.149	-	-		
	AJCC II	59	37			-	-		
	AJCC III	60	37			-	-		
	AJCC IV	6	0			-	-		
LUAD	AJCC I	126	167	6.203	0.102	-	-		
	AJCC II	62	69			-	-		
	AJCC III	36	56			-	-		
	AJCC IV	18	10			-	-		
KIRP	Female	51	33	0.094	0.759	-	-		
	Male	137	96			-	-		
READ	Female	22	27	1.653	0.199	-	-		
	Male	30	22			-	-		

Notes: $^{*}p < 0.05$.
