# Supplementary material for: Gene S-phase kinase associated protein 2 is a novel prognostic marker in human neoplasms
Source: BMC Med Genomics. 2023 Jun 12;16:128. doi: 10.1186/s12920-023-01561-4 (PMC10259050; doi:10.1186/s12920-023-01561-4)
Supplement: Supplementary file 15 — Supplementary Material 15. KEGG results based on gene set enrichment analysis in this study [file 12920_2023_1561_MOESM15_ESM.pdf]

Supplementary Material 15. KEGG results based on gene set enrichment analysis in this study.

Description	EnrichmentScore	NES	P-value	Cancer
AUTOIMMUNE THYROID DISEASE	-0.97364	-1.3741	0.009259	ACC
GRAFT VERSUS HOST DISEASE	-0.97346	-1.34026	0.019048	ACC
ALLOGRAFT REJECTION	-0.97364	-1.34756	0.028302	ACC
ASTHMA	-0.98415	-1.34672	0.030612	ACC
AUTOIMMUNE THYROID DISEASE	-0.97364	-1.3741	0.009259	ACC
GRAFT VERSUS HOST DISEASE	-0.97346	-1.34026	0.019048	ACC
ALLOGRAFT REJECTION	-0.97364	-1.34756	0.028302	ACC
ASTHMA	-0.98415	-1.34672	0.030612	ACC
OLFACTORY TRANSDUCTION	-0.85323	-1.51478	0.002907	BRCA
NATURAL KILLER CELL MEDIATED CYTOTOXICITY	0.909408	1.322376	0.015649	BRCA
COMPLEMENT AND COAGULATION CASCADES	-0.92927	-1.3318	0.047368	BRCA
CARDIAC MUSCLE CONTRACTION	-0.93159	-1.32385	0.018018	CESC
NEUROACTIVE LIGAND RECEPTOR INTERACTION	-0.80206	-1.32687	0.03271	CESC
ASCORBATE AND ALDARATE METABOLISM	0.97352	1.230175	0.033206	CESC
RETINOL METABOLISM	0.951069	1.186691	0.01697	CHOL
METABOLISM OF XENOBIOTICS BY CYTOCHROME P450	0.950015	1.259225	0.029337	COAD
STEROID HORMONE BIOSYNTHESIS	0.95461	1.252661	0.040558	COAD
RIBOSOME	-0.8838	-1.50876	0.014286	DLBC
OLFACTORY TRANSDUCTION	0.880402	1.443498	0.002821	ESCA
CALCIUM SIGNALING PATHWAY	-0.84863	-1.35645	0.040625	ESCA
LEUKOCYTE TRANSENDOTHELIAL MIGRATION	-0.89031	-1.31346	0.042945	ESCA
AUTOIMMUNE THYROID DISEASE	-0.96345	-1.37685	0.007299	GBM
GRAFT VERSUS HOST DISEASE	-0.95676	-1.32752	0.022901	GBM
HEMATOPOIETIC CELL LINEAGE	-0.88865	-1.32064	0.027397	GBM
PHENYLALANINE METABOLISM	0.976264	1.204831	0.035348	GBM
CYTOKINE CYTOKINE RECEPTOR INTERACTION	-0.70193	-1.33162	0.038835	GBM
MATURITY ONSET DIABETES OF THE YOUNG	0.968958	1.167029	0.039046	HNSCC
OLFACTORY TRANSDUCTION	0.898137	1.127401	0.002	KIRC
NEUROACTIVE LIGAND RECEPTOR INTERACTION	0.89758	1.124935	0.006006	KIRC

OXIDATIVE PHOSPHORYLATION	-0.76326	-1.31387	0.039216	KIRP
OLFACTORY TRANSDUCTION	0.907748	1.101719	0.003	LAML
TASTE TRANSDUCTION	0.946562	1.132984	0.022704	LAML
OLFACTORY TRANSDUCTION	0.93758	1.205182	0.001002	LIHC
RETINOL METABOLISM	-0.94543	-1.38977	0.016667	LIHC
MATURITY ONSET DIABETES OF THE YOUNG	0.990782	1.246842	0.001241	LUSC
OLFACTORY TRANSDUCTION	0.901001	1.295344	0.004391	LUSC
HEMATOPOIETIC CELL LINEAGE	-0.93989	-1.37417	0.006452	LUSC
COMPLEMENT AND COAGULATION CASCADES	-0.94883	-1.40005	0.019608	LUSC
MATURITY ONSET DIABETES OF THE YOUNG	0.994715	1.246979	0.001325	MESO
MATURITY ONSET DIABETES OF THE YOUNG	0.98561	1.202702	0.004246	OV
STEROID HORMONE BIOSYNTHESIS	0.934223	1.14454	0.039583	OV
OLFACTORY TRANSDUCTION	-0.88061	-1.74651	0.008696	PAAD
MATURITY ONSET DIABETES OF THE YOUNG	-0.97785	-1.33513	0.040541	PAAD
NEUROACTIVE LIGAND RECEPTOR INTERACTION	0.920698	1.220598	0.002043	PRAD
CYTOKINE CYTOKINE RECEPTOR INTERACTION	0.907662	1.20153	0.003067	PRAD
OLFACTORY TRANSDUCTION	-0.96298	-1.66717	0.002653	READ
PENTOSE AND GLUCURONATE INTERCONVERSIONS	0.971809	1.278174	0.038251	READ
METABOLISM OF XENOBIOTICS BY CYTOCHROME P450	0.939575	1.293268	0.039706	READ
RIBOSOME	-0.92517	-1.43731	0.005376	SARC
COMPLEMENT AND COAGULATION CASCADES	-0.9156	-1.34654	0.010638	SARC
ALLOGRAFT REJECTION	-0.95328	-1.31569	0.038889	SARC
ANTIGEN PROCESSING AND PRESENTATION	-0.87835	-1.34295	0.042328	SARC
CHEMOKINE SIGNALING PATHWAY	-0.7712	-1.36777	0.047619	SARC
ASTHMA	-0.97392	-1.40582	0.011765	SKCM
AUTOIMMUNE THYROID DISEASE	-0.9144	-1.36577	0.046154	SKCM
CALCIUM SIGNALING PATHWAY	-0.86245	-1.34426	0.032037	STAD
MAPK SIGNALING PATHWAY	-0.80868	-1.35562	0.043779	STAD
RETINOL METABOLISM	0.945112	1.259351	0.010376	TGCT
AUTOIMMUNE THYROID DISEASE	-0.95357	-1.33276	0.016529	TGCT
GRAFT VERSUS HOST DISEASE	-0.95391	-1.31161	0.033755	TGCT

ALLOGRAFT REJECTION	-0.9557	-1.30801	0.042553	TGCT
OLFACTORY TRANSDUCTION	0.900973	1.28478	0.00102	THYM
ASTHMA	-0.9507	-1.38864	0.019417	UCEC
OLFACTORY TRANSDUCTION	0.874466	1.186844	0.021212	UCEC
LINOLEIC ACID METABOLISM	-0.93489	-1.38775	0.028302	UCEC
OLFACTORY TRANSDUCTION	0.930535	1.491466	0.001161	UCS
CARDIAC MUSCLE CONTRACTION	0.942061	1.274835	0.019034	UCS
MATURITY ONSET DIABETES OF THE YOUNG	0.977685	1.228396	0.024963	UCS
