# Supplementary material for: Gene S-phase kinase associated protein 2 is a novel prognostic marker in human neoplasms
Source: BMC Med Genomics. 2023 Jun 12;16:128. doi: 10.1186/s12920-023-01561-4 (PMC10259050; doi:10.1186/s12920-023-01561-4)
Supplement: Supplementary file 16 — Supplementary Material 16. A summary of KEGG results based on gene set enrichment analysis in this study [file 12920_2023_1561_MOESM16_ESM.pdf]

**Supplementary Material 16.** A summary of KEGG results based on gene set enrichment analysis.

| KEGG signaling pathways | Observed in cancer types |
|---|---|
| OLFACTORY TRANSDUCTION | 11 cancers |
| MATURITY ONSET DIABETES OF THE YOUNG | 6 cancers |
| AUTOIMMUNE THYROID DISEASE | 5 cancers |
| ALLOGRAFT REJECTION | 4 cancers |
| ASTHMA | 4 cancers |
| GRAFT VERSUS HOST DISEASE | 4 cancers |
| COMPLEMENT AND COAGULATION CASCADES | 3 cancers |
| NEUROACTIVE LIGAND RECEPTOR INTERACTION | 3 cancers |
| RETINOL METABOLISM | 3 cancers |
| CALCIUM SIGNALING PATHWAY | 2 cancers |
| CARDIAC MUSCLE CONTRACTION | 2 cancers |
| CYTOKINE CYTOKINE RECEPTOR INTERACTION | 2 cancers |
| HEMATOPOIETIC CELL LINEAGE | 2 cancers |
| METABOLISM OF XENOBIOTICS BY CYTOCHROME P450 | 2 cancers |
| RIBOSOME | 2 cancers |
| STEROID HORMONE BIOSYNTHESIS | 2 cancers |
| ANTIGEN PROCESSING AND PRESENTATION | 1 cancer |
| ASCORBATE AND ALDARATE METABOLISM | 1 cancer |
| CHEMOKINE SIGNALING PATHWAY | 1 cancer |
| LEUKOCYTE TRANSENDOTHELIAL MIGRATION | 1 cancer |
| LINOLEIC ACID METABOLISM | 1 cancer |
| MAPK SIGNALING PATHWAY | 1 cancer |
| NATURAL KILLER CELL MEDIATED CYTOTOXICITY | 1 cancer |
| OXIDATIVE PHOSPHORYLATION | 1 cancer |
| PENTOSE AND GLUCURONATE INTERCONVERSIONS | 1 cancer |
| PHENYLALANINE METABOLISM | 1 cancer |
| TASTE TRANSDUCTION | 1 cancer |
